# Supplementary figures and images for: MxB inhibits long interspersed element type 1 retrotransposition
Source: PLoS Genet. 2022 Feb 16;18(2):e1010034. doi: 10.1371/journal.pgen.1010034 (PMC8849481; doi:10.1371/journal.pgen.1010034)

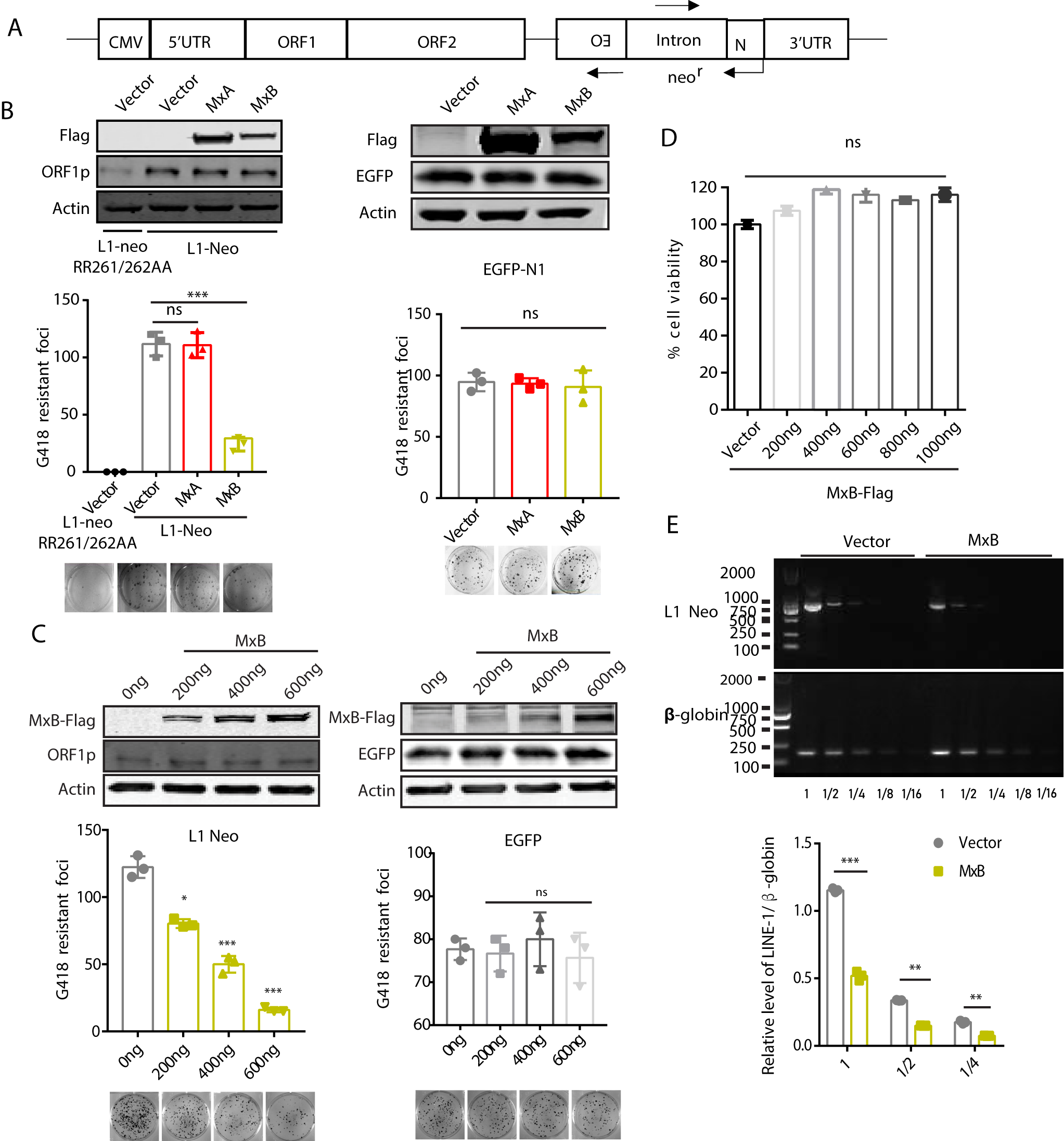

Supplement: S1 Fig — (A) Illustration of the L1-neoRT reporter cassette. CMV-L1-neoRT reporter DNA contains the complete human LINE-1 DNA and a neomycin resistance gene as a reporter of LINE-1 retrotransposition and a CMV promoter before 5’UTR. (B) HeLa cells were transfected with CMV-L1-neoRT, defective L1-ORF1 (RR261/262AA) or pEGFP-N1 (carrying neomycin resistant gene) DNA together with MxA or MxB DNA. G418-resistant cell colonies were scored and results of three independent experiments are presented in the bar graph. Images of representative colony assays are shown. Ectopic expression of MxA-Flag or MxB-Flag was examined by Western blot. (C) HeLa cells were transfected with CMV-L1-neoRT or pEGFP-N1 DNA together with increasing doses of MxB DNA. Results of three independent experiments are presented in the bar graphs. (D) Cell viability of HeLa cells transfected with different doses of MxB-Flag plasmid DNA for 48h. (E) HEK293T cells were co-transfected with the CMV-L1-neoRT DNA and MxB DNA. Levels of the newly synthesized LINE-1 DNA were determined by semi-quantitative PCR. Levels of β-globin DNA were measured as internal controls. Intensities of DNA bands were quantified, the results are summarized in the bar graph (mean ± SEM; paired t-test). *, P<0.05; **, P< 0.01; ***, P<0.001. (TIF) [file pgen.1010034.s001.tif]

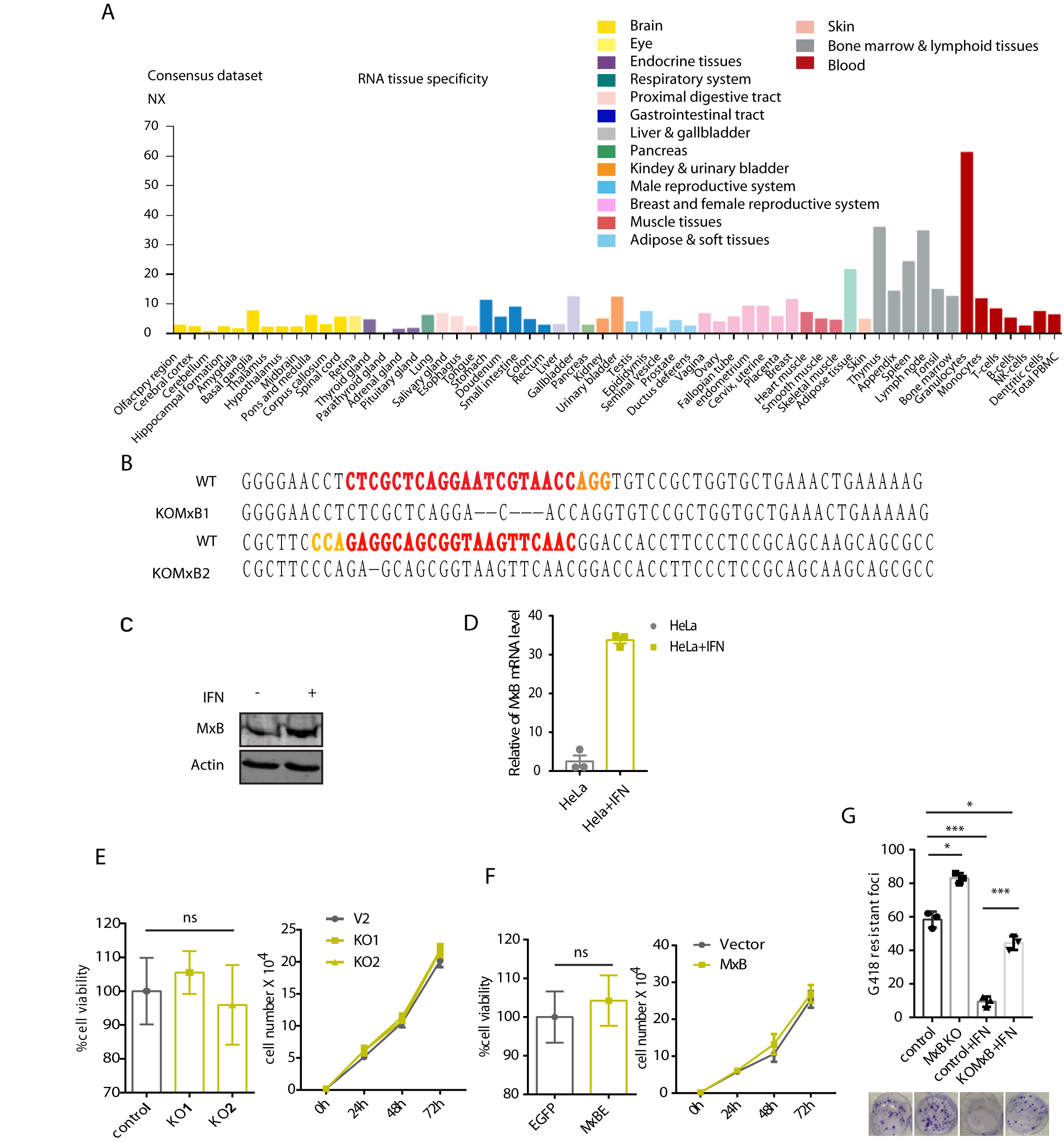

Supplement: S2 Fig — (A) MxB RNA levels in human tissues. Consensus Normalized eXpression (NX) levels for 55 tissue types and 6 blood cell types, created by combining the data from the three transcriptomics datasets (HPA, GTEx and FANTOM5), reference from www.proteinatlas.org. (B) The two guide RNA sequences are shown in red letters. The protospacer adjacent motifs (PAMs) are shown in orange letters. The mutated MxB sequences at the gRNA target sites are presented for each cell clone which was selected and used in this study. (C, D) Endogenous MxB protein and its mRNA level were determined with Western blot and RT-qPCR after stimulation with IFNα1 (25 ng/mL) for 16 hours. (E, F) Cell viability and cell growth rate of MxB knockout cell lines (E) and stable MxB-expressing cell line (F) were detected by cell-counting kit-8. (G) Colony assay was performed with MxB knockout or control cell lines which were transfected with CMV-L1-neoRT plasmid (500 ng) for 24 hours and treated with IFNα1 (2.5 ng/mL) for 24 hours. The results was presented in the bar graph (mean ± SEM; paired t-test). ns, not significant. *, P<0.1; **; P<0.01; ***, P<0.001. (TIF) [file pgen.1010034.s002.tif]

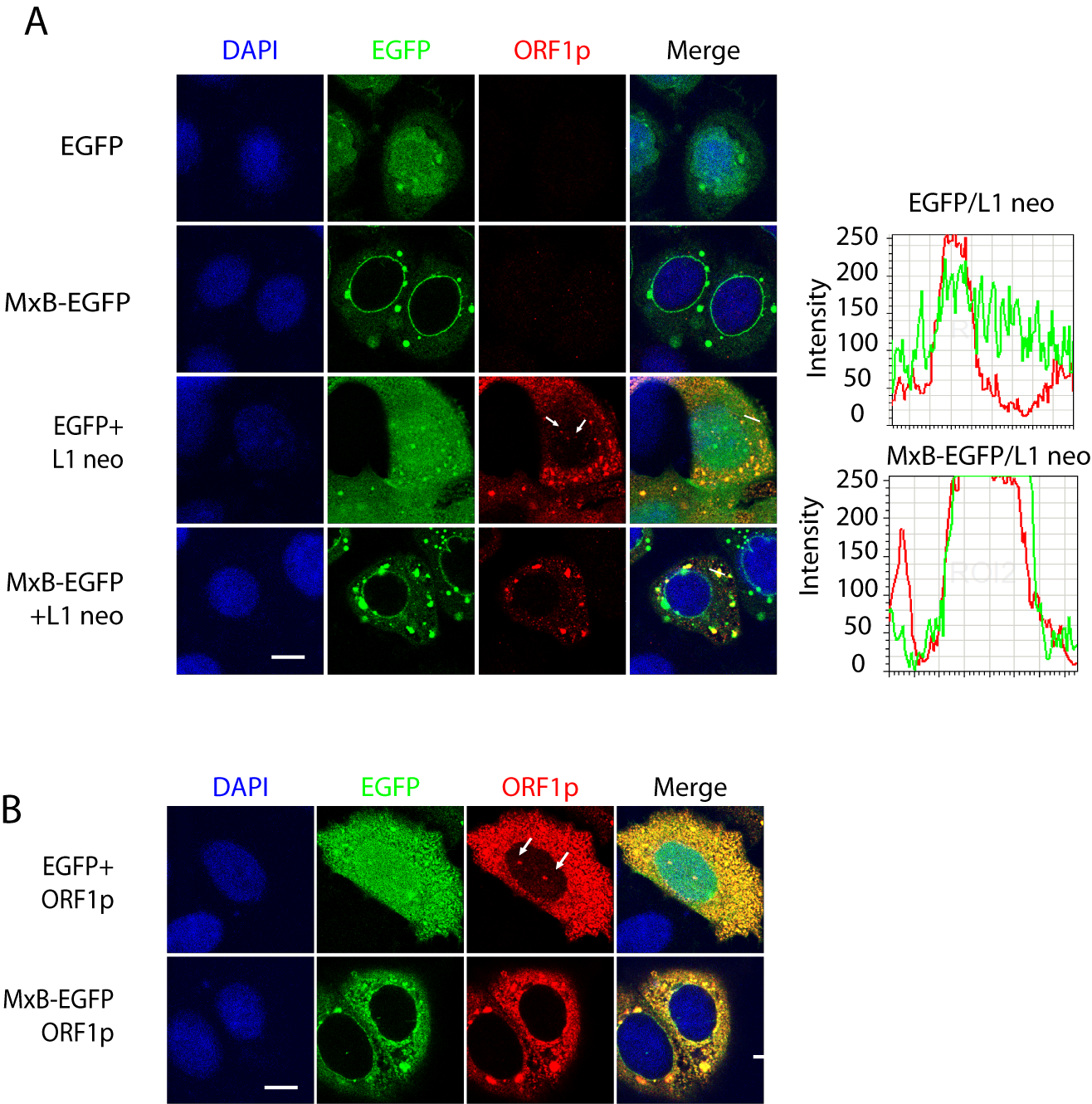

Supplement: S3 Fig — (A, B) HeLa cells were co-transfected with MxB-EGFP and CMV-L1-neoRT reporter (A) or ORF1p-Flag DNA (B). Distribution of MxB and ORF1p was examined by immunofluorescence microscopy. The white arrows showed nuclear ORF1p. Fluorescence intensity analysis was performed at the position which indicated by white lines in merge panel to quantify the co-localization of EGFP or MxB-EGFP with ORF1p (A). Co-localization analyzed with fluorescence intensity used software from LAS AF (Leica). Scale bar, 10 μm. (TIF) [file pgen.1010034.s003.tif]

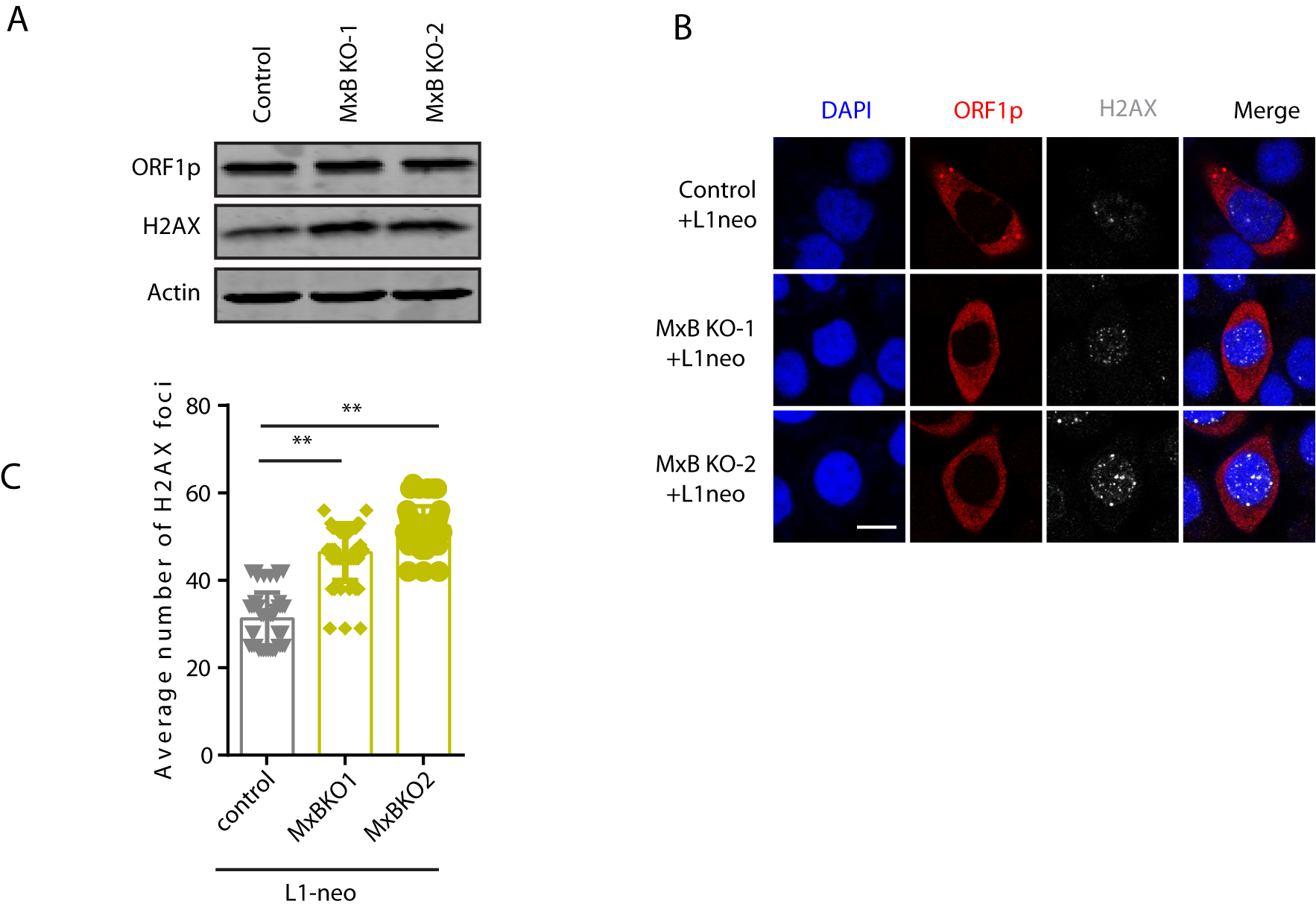

Supplement: S4 Fig — (A) Western blot to detect γH2AX in the control or MxB knockout HeLa cells which were transfected with CMV-L1-neoRT plasmid. (B, C) Detection of γH2AX foci in the control and MxB knockout cells transfected with CMV-L1-neoRT plasmid. The γH2AX foci were scored in 50 cells, the results are presented in (C) (mean ± SEM; paired t-test). ** indicates P<0.01; ***, P<0.001. Scale bar, 10 μm. (TIF) [file pgen.1010034.s004.tif]

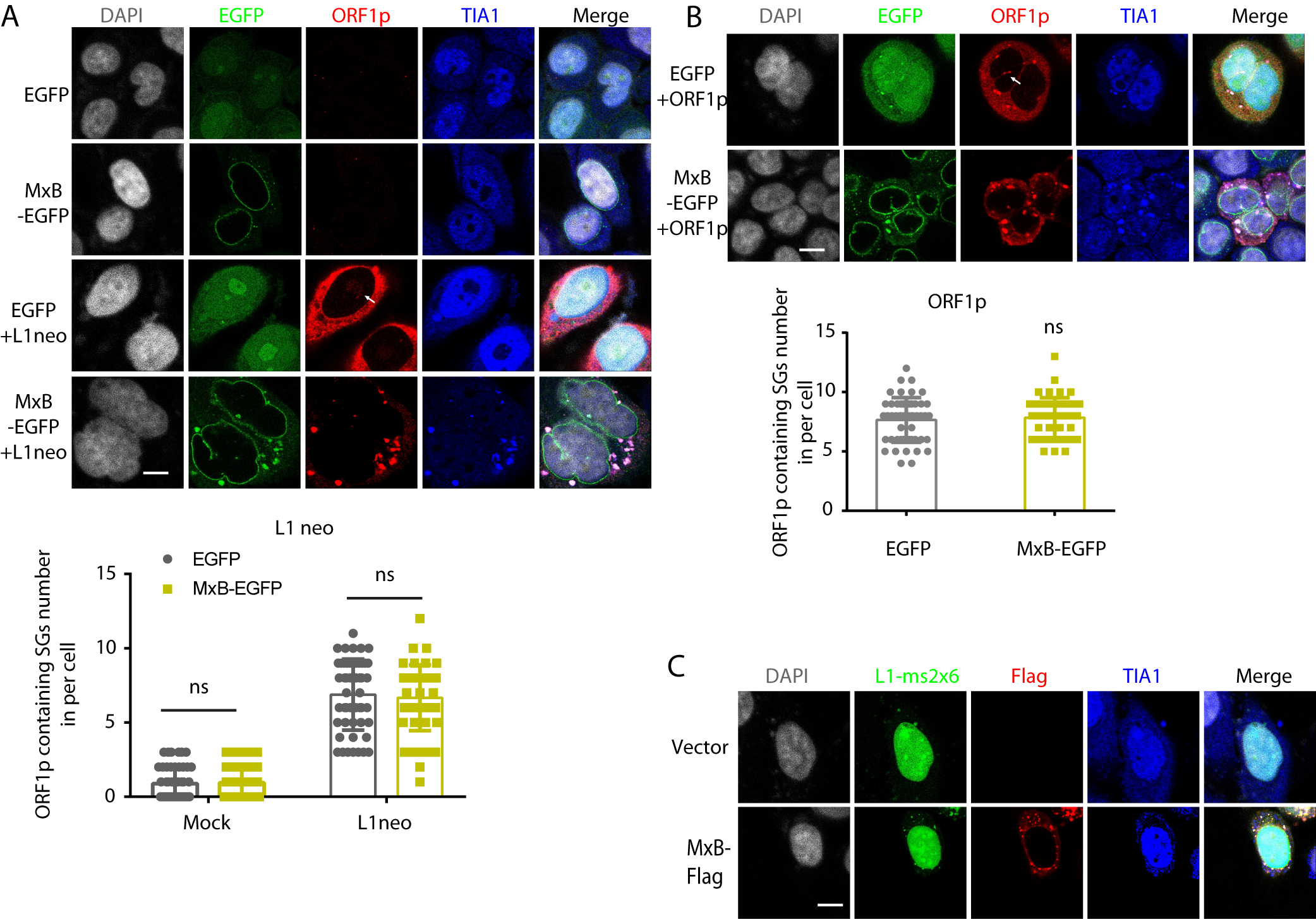

Supplement: S5 Fig — (A, B) Co-localization of stably expressed MxB-EGFP with ORF1p and TIA1 in HeLa cells. ORF1p was either expressed from the transfected CMV-L1-neoRT DNA (500ng) (A) or ORF1p vector DNA (500ng) (B) for 24 hours. White arrows indicate nuclear ORF1p. ORF1p/TIA1-containing SGs were scored in more than 50 cells for each treatment. The average number of ORF1p-containing SGs per cell is presented in the bar graph (mean ± SEM; paired t-test). ns, no significant. (C) HeLa cells were co-transfection with MxB-Flag (500 ng), LINE-1-ms2x6 (750 ng) and MS2-GFP (250 ng) plasmid DNA. Subcellular location of LINE-1 RNA was indicated by the binding of MS2-GFP to the 6 MS2-binding sites in the LINE-1 RNA. Scale bar, 10 μm. (TIF) [file pgen.1010034.s005.tif]

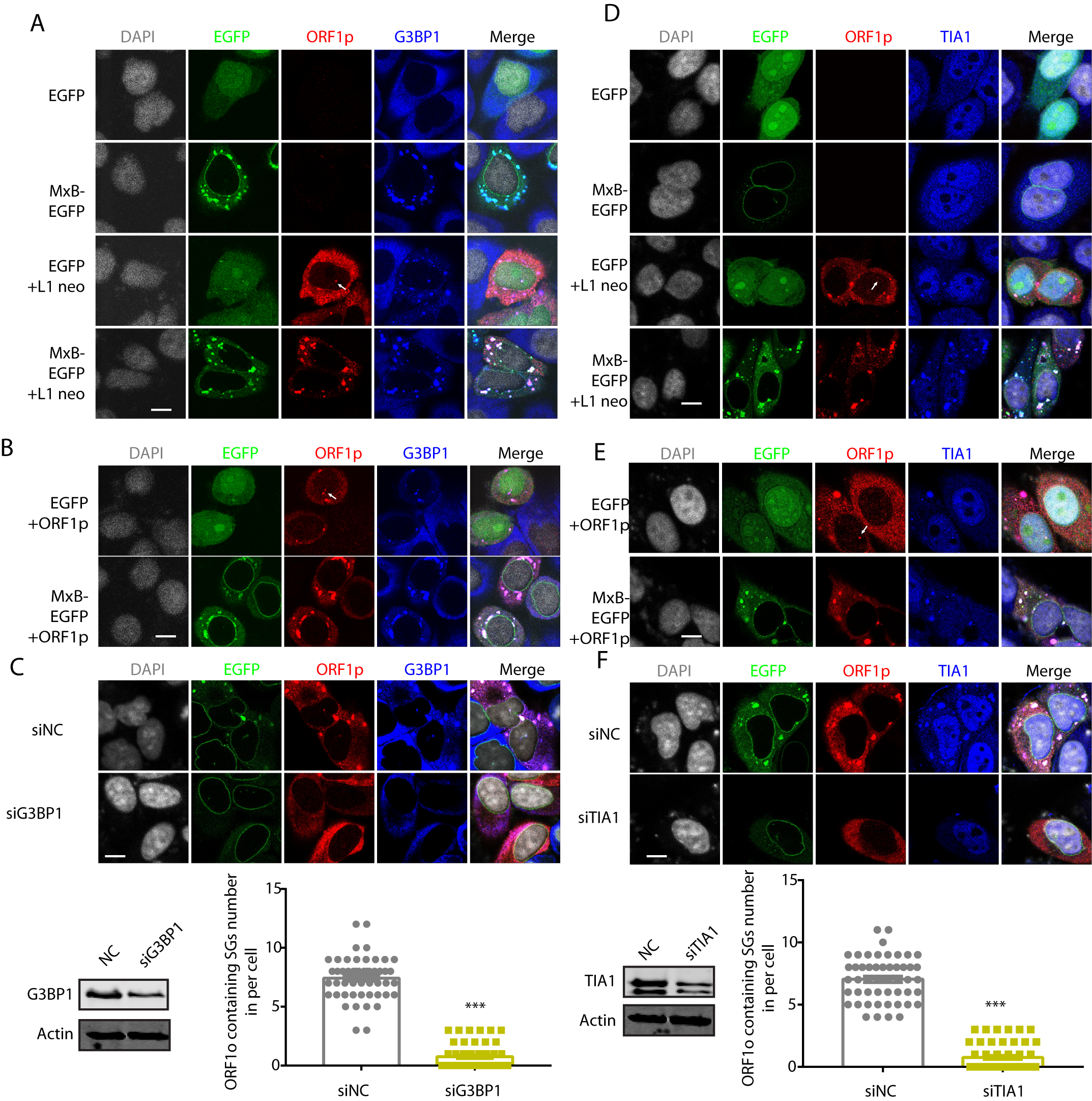

Supplement: S6 Fig — (A, B) Immunofluorescence microscopy to detect ORF1p, G3BP1 and MxB in HeLa cells co-transfected with MxB-EGFP (500ng) and CMV-L1-neoRT DNA (500ng) (A) or the ORF1p-Flag DNA (500ng) (B) for 24 hours. The white arrows indicate nuclear ORF1p. (C) The MxB-EGFP stably expressing HeLa cell lines were transfected with siRNAs targeting G3BP1 and then transfected with CMV-L1-neoRT DNA (500ng). Immunofluorescence was performed to detect ORF1p, G3BP1 and MxB 24 hours after transfection. Expression of G3BP1 was examined by Western blot. Scale bar, 10 μm. (D, E) Immunofluorescence was performed to detect ORF1p, TIA1 and MxB in HeLa cells co-transfected with MxB and CMV-L1-neoRT DNA (500ng) (C) or the ORF1p-Flag DNA (500ng) (D). The white arrows indicate nuclear ORF1p. (F) MxB-EGFP stably expressing HeLa cells were transfected with siRNAs targeting TIA1 and then transfected with CMV-L1-neoRT DNA. Immunofluorescence was performed to detect ORF1p, TIA1 and MxB 24 hours post transfection. Expression of TIA1 was examined by Western blot. ORF1p-containing SGs were scored in more than 50 cells for each treatment. The average number of ORF1p-containing SGs per cell is presented in the bar graph (mean ± SEM; paired t-test) in (C and F). ***, P<0.001. Scale bar, 10 μm. (TIF) [file pgen.1010034.s006.tif]

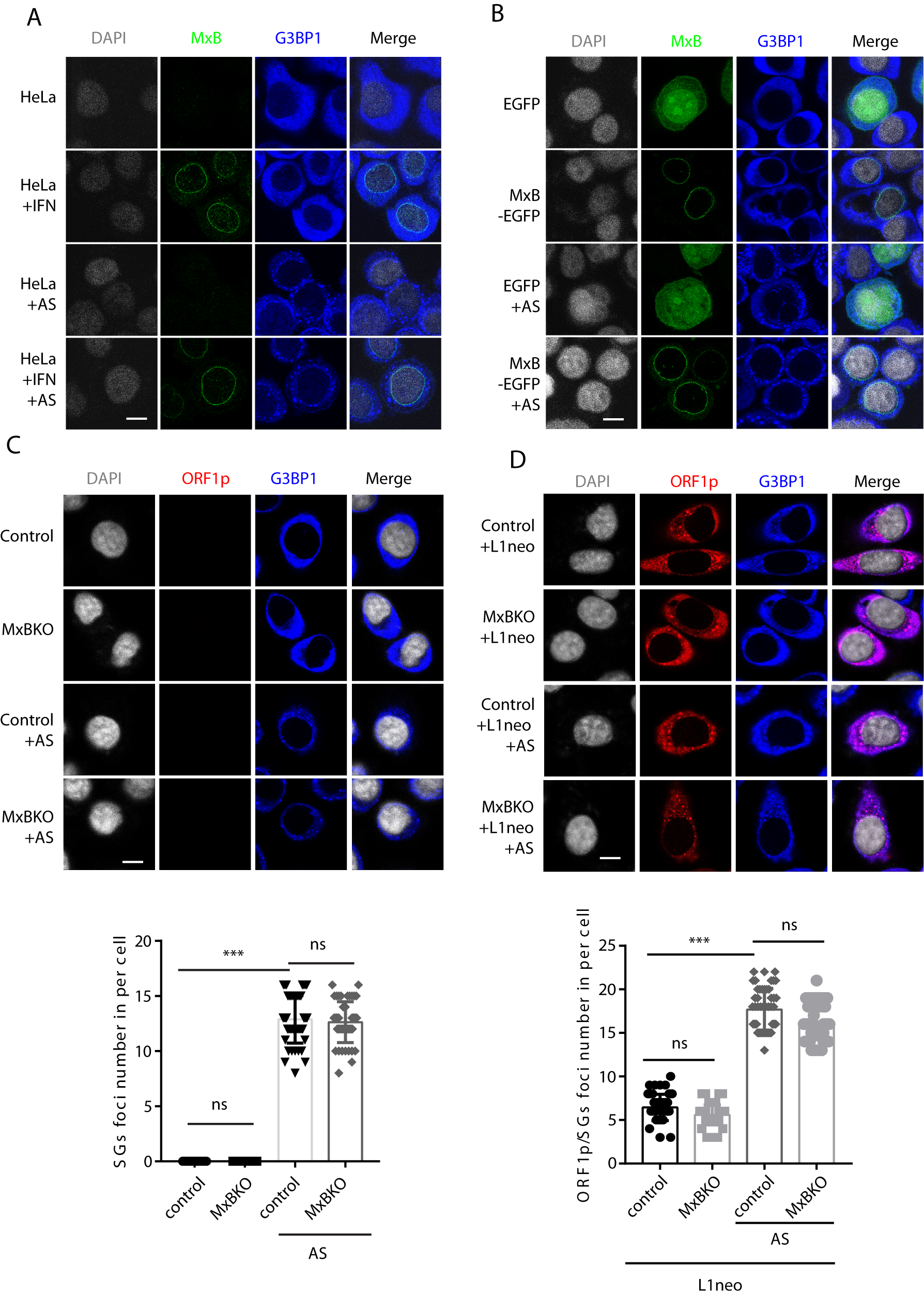

Supplement: S7 Fig — (A) HeLa cells were treated with IFN-α (25 ng/mL) for 24 hours, followed by exposure to arsenite (500 μM) for 30 min. G3BP1 and MxB were detected by immunostaining and fluorescence microscopy. (B) HeLa cells which stably express MxB-EGFP were treated with arsenite (500 μM) for 30 min. G3BP1 and MxB were detected by immunofluorescence microscopy. (C, D) MxB knockout cells were transfected CMV-L1-neoRT DNA (D) or vector control (C) for 24 hours, and then treated with arsenite (500 μM) for 30 min. SGs number was scored in more than 50 cells for each treatment, the results of three independent experiments are presented in the bar graph. The average number of SGs per cell is presented in the bar graph (mean ± SEM; paired t-test) in (C and D). ns, not significant. ***, P<0.001. Scale bar, 10 μm. (TIF) [file pgen.1010034.s007.tif]

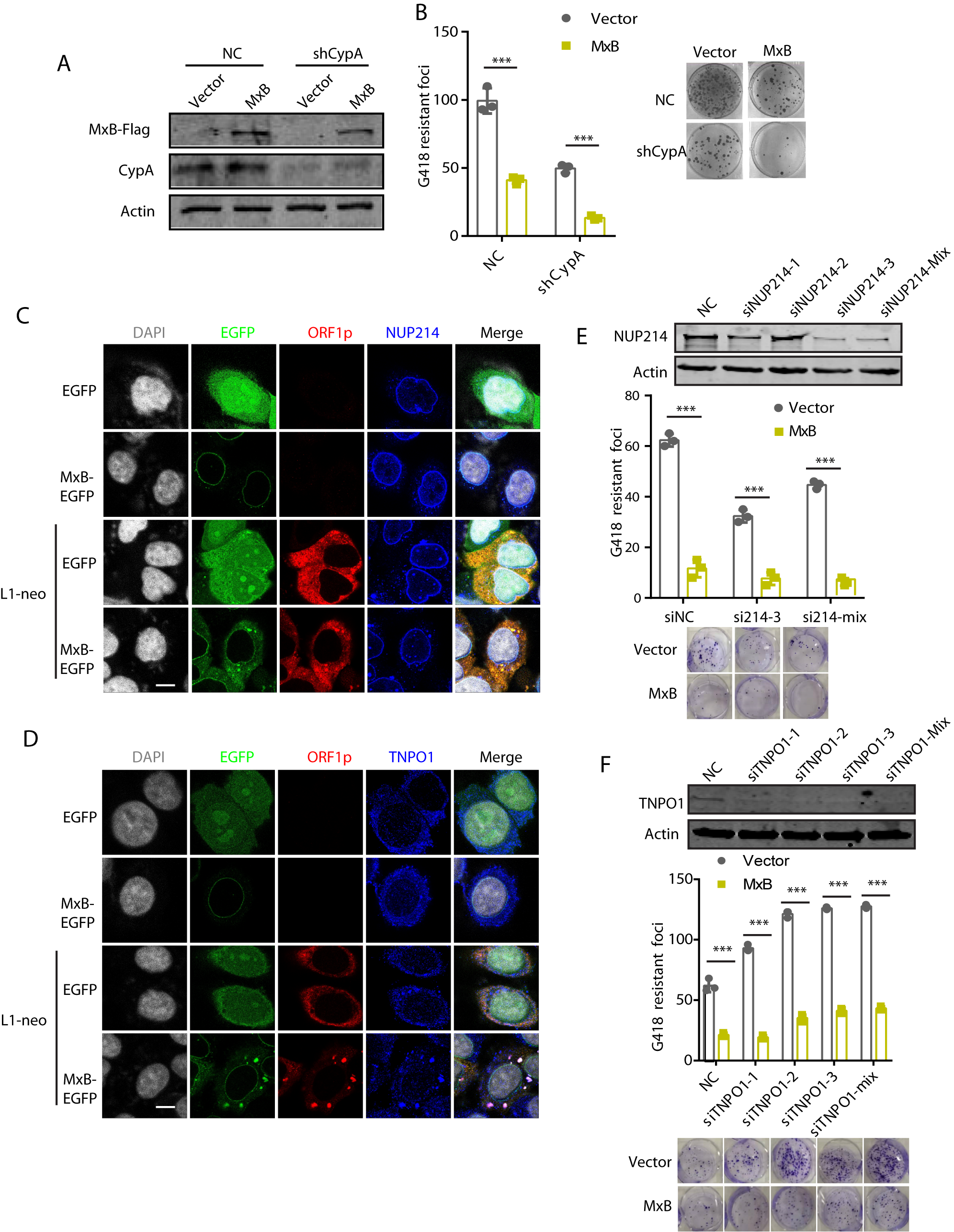

Supplement: S8 Fig — (A, B) CypA was knocked down before MxB and CMV-L1-neoRT were co-transfected into HeLa cells. Western blot was performed 48 hours post transfection to measure CypA and MxB levels (A). Meanwhile, G418 was added at 48 hours post transfection. G418 resistant colonies were scored and results of three independent experiments are presented in the bar graph (B). (C, D) Endogenous NUP214 (C), TNPO1 (D), CMV-L1-neoRT ORF1p and stably expressed MxB-EGPF were detected by immunofluorescence. (E, F) Effect of knocking down endogenous NUP214 (E) or TNPO1 (F) on MxB inhibition of CMV-L1-neoRT in HeLa cells. Results of three independent experiments are presented in the bar graph (mean ± SEM; paired t-test). Images of representative colony assays are shown. Expression of NUP214 and TNPO1 was examined by Western blot. ns, not significant. ** indicates P<0.01; ***, P<0.001. Scale bar, 10 μm. (TIF) [file pgen.1010034.s008.tif]

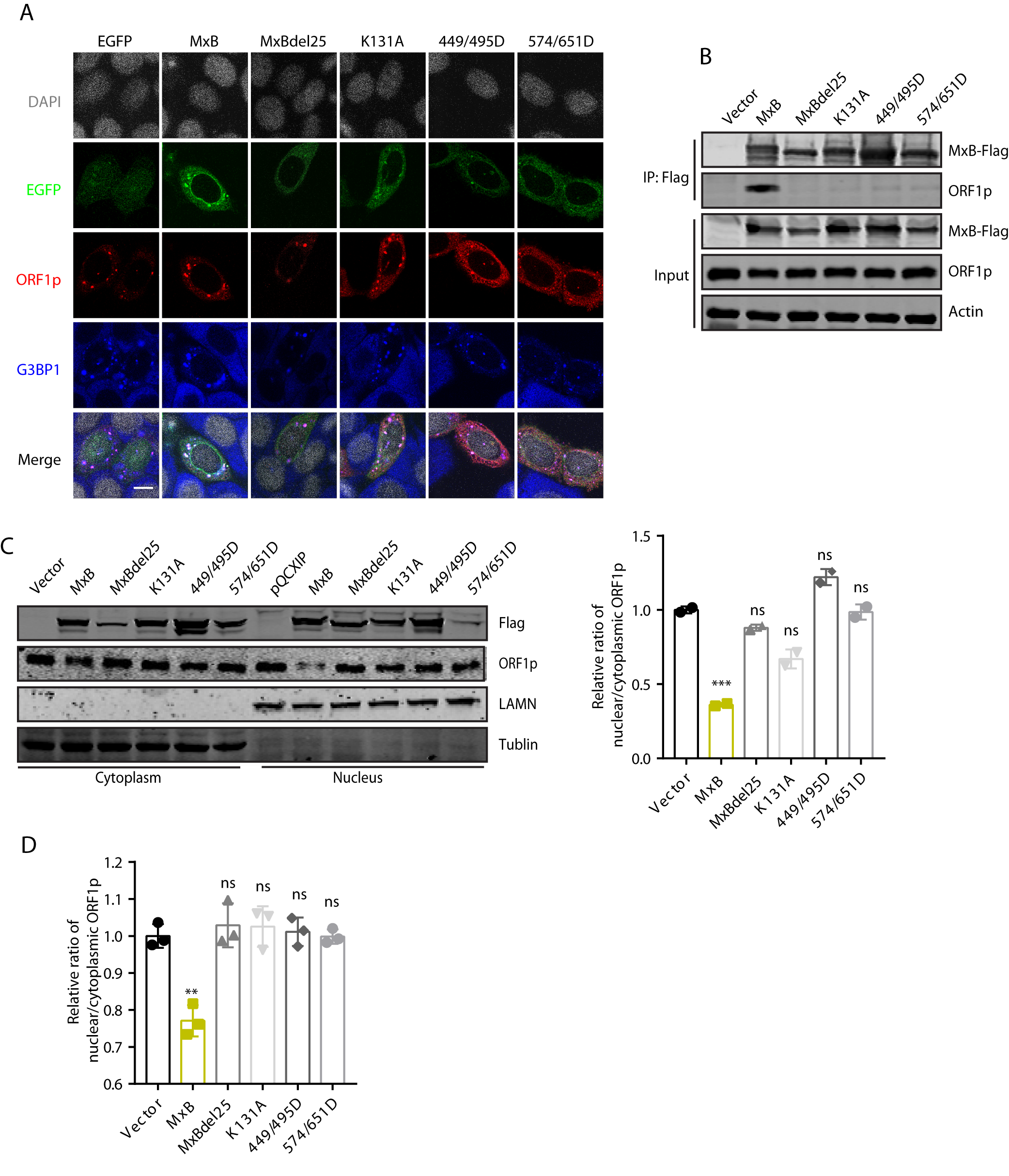

Supplement: S9 Fig — (A) Immunofluorescence microscopy analysis of ORF1p, G3BP1 and MxB in HeLa cells co-transfected with CMV-L1-neoRT and MxB or its mutants. Scale bar, 10 μm. (B) 293T cells were co-transfected with CMV-L1-neoRT DNA and MxB-Flag or its mutants. Immunoprecipitation was performed with anti-Flag antibody 48 hours post transfection. Presence of ORF1p in the precipitated materials was detected by Western blot. (C) HeLa cells were co-transfected with CMV-L1-neoRT and MxB or its mutant DNA. Nuclear and cytoplasmic fractions were prepared and further examined in Western blot to determine the levels of ORF1p. The bar graph at the S9C right was the quantification of the immunoblot. (D) The Operetta High-Content Screen system (PerkinElmer) was utilized to determine the ratios of the nuclear and cytoplasmic ORF1p. The results are summarized in the bar graph (mean ± SEM; paired t-test). ns, not significant. **, P<0.01; ***, P<0.001. (TIF) [file pgen.1010034.s009.tif]

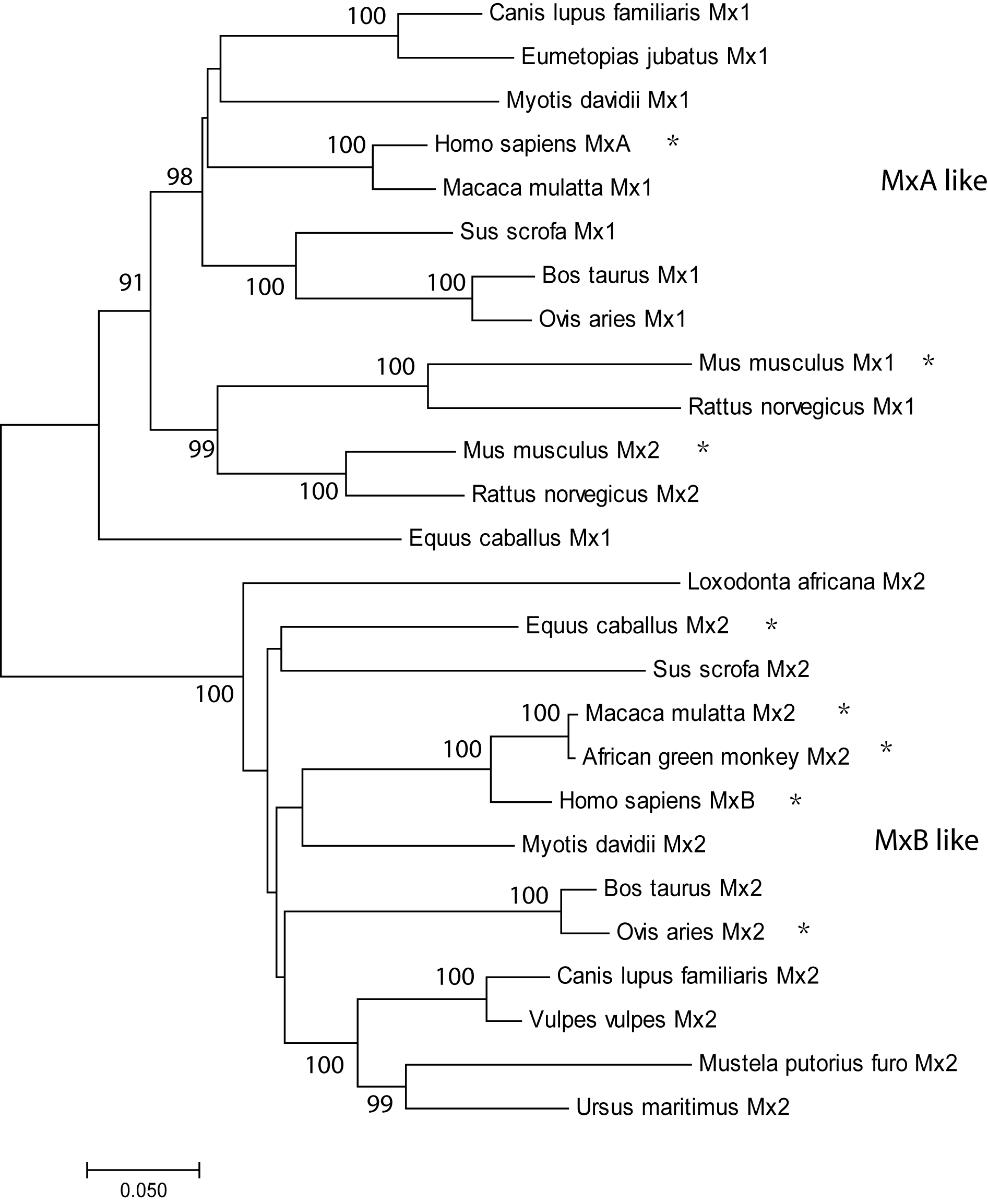

Supplement: S10 Fig — Neighbor-Joining method was used for the evolution analysis. Orthologous Mx2 sequences were acquired from Genbank. The protein sequences were aligned using Clustal Omega and converted to a codon alignment using MEGA 7. The Neighbor-Joining tree was calculated in MEGA 7 with 1,000 bootstrap replicates. Bootstrap values (>70) were also tested. Mx genes were selected from MxA-like and MxB-like major clades. “*” indicates the Mx genes that were investigated in this study for inhibiting LINE-1 retrotransposition. (TIF) [file pgen.1010034.s010.tif]
