# Supplementary material for: MxB inhibits long interspersed element type 1 retrotransposition
Source: PLoS Genet. 2022 Feb 16;18(2):e1010034. doi: 10.1371/journal.pgen.1010034 (PMC8849481; doi:10.1371/journal.pgen.1010034)
Supplement: S1 Table — (DOCX) [file pgen.1010034.s011.docx]

**Table 1 Primers and RNAs used in this study**

| Primers’name | Sequence |
| --- | --- |
| 5’UTRF | 5’-AGAGAGCAGTGGTTCTCCCAGCACG-3’ |
| 5’UTRR | 5’-CTGTTGGAATACCCTGCCGTGTGAG-3’ |
| ORF1pF | 5’-GCAGTTCCTCACCAGCAACAGAACA-3’ |
| ORF1pR | 5’-GCCTTTGGTTTGAATGTCCTCCCG-3’ |
| ORF2pF | 5’-TATTCAGGAAACCCATCTCACGTGC-3’ |
| ORF2pR | 5’-GGGTGCTCCTGTATTGGGTGCATAA-3’ |
| GAPDH-F | 5’-GCAAATTCCATGGCACCGT-3’ |
| GAPDH-R | 5’-GCAAATTCCATGGCACCGT-3’ |
| L1cDNAF | 5’-CAGTTCGGCTGGCGCGAGGCC-3’ |
| L1cDNAR | 5’-CAGTTCCGCCCATTCTCCG-3 |
| β-globinF | 5’-TATTGGTCTCCTTAAACCTGTCTTG-3’ |
| β-globinR | 5’-CTGACACAACTGTGTTCACTAGC-3’ |
| mL1_TfF | 5’-CAGCGGTCGCCATCTTG-3’ |
| mL1_TfR | 5’-CACCCTCTCACCTGTTCAGACTAA-3’ |
| mL1_GfF | 5’-CTCCTTGGCTCCGGGACT-3’ |
| mL1_GfR | 5’-CAGGAAGGTGGCCGGTTGT-3’ |
| mL1_AF | 5’-GGATTCCACACGTGATCCTAA-3’ |
| mL1_AR | 5’-TCCTCTATGAGCAGACCTGGA-3’ |
| mSineF | 5’-GTGGCGCACGCCTTTAATC-3’ |
| mSineR | 5’-GACAGGGTTTCTCTGTGTAG-3’ |
| mRrm2-F | 5’-CCGAGCTGGAAAGTAAAGCG-3’ |
| mRrm2-R | 5’-ATGGGAAAGACAACGAAGCG-3’ |
| **sgRNA** | **Sequence** |
| MxB gRNA1 | 5’-CTCGCTCAGGAATCGTAACCAGG-3’ |
| MxB gRNA2 | 5’-CCAGAGGCAGCGGTAAGTTCAAC-3’ |
| **siRNA name** | **Sequence** |
| siG3BP1-1 | 5’-GGGCTTCTCTCTAACAACA-3’ |
| siG3BP1-2 | 5’-GGACAAATCAGAGCTTAAA-3’ |
| siTIA1-1 | 5’-CCGCTCCAAAGAGTACATA-3’ |
| siTIA1-2 | 5’-GGCTAACAGAACAACTAAT-3’ |
| siNUP214-1 | 5’-GCACAATGCTTGCCACGAA-3’ |
| siNUP214-2 | 5’-GGACAGTCTTCTCCCAACA-3’ |
| siNUP214-3 | 5’-GTGGAAAGATTCAGATCCT-3’ |
| siTNPO1-1 | 5’-GAGCACTTGTGATGTTGCT-3’ |
| siTNPO1-2 | 5’-CACCAAAGACGTTGTTAGA-3’ |
| siTNPO1-3 | 5’-GACTCTTAGCCGCTATGCA-3’ |
