# Supplementary material for: MxB inhibits long interspersed element type 1 retrotransposition
Source: PLoS Genet. 2022 Feb 16;18(2):e1010034. doi: 10.1371/journal.pgen.1010034 (PMC8849481; doi:10.1371/journal.pgen.1010034)
Supplement: Attachment — Submitted filename: responses to reviwers PGENETICS-D-21-01057 R1.docx [file pgen.1010034.s012.docx]

**Manuscript Number: PGENETICS-D-21-01057**

**Title:** **MxB inhibits Long interspersed element 1 retrotransposition**

**Journal: plos genetics**

Dear Editor and Reviewers:

Thank you for careful reading of our manuscript and constructive comments. We have now addressed all your comments, and revised the manuscript accordingly. The changes are included in the revised manuscript. Responses to your individual comments are listed below.

***Reviewer #1:***

*In this work Huang and colleagues investigate the inhibitory activity of the interferon stimulated protein MXB on the transposable element LINE-1. Authors describe how ectopically expressed MXB inhibits integration of LINE-1 in HeLa cells. Authors present evidence for the interaction and colocalization of MXB and ORF1P, in a complex that also colocalize with stress granule marker G3BP1. For inhibition to happen, MXB requires its N-terminal nuclear envelope localization signal, its GTPase and oligomerization activities, as well as the presence of stress granules components G3BP1 and TIA1. In a final set of experiments, they broaden the spectrum of MXB inhibition to other transposable elements such as IAP and Mus D.
This paper is interesting because it further widens the functions of MXB (MX2) as an "antiviral" factor, addressing its activity against retrotransposon elements. The notion that MXB itself has a potent inhibitory activity on restricting LINE-1 nuclear transport could be specially interesting for tissues with a “high” basal levels of MXB (such as granulocytes). In addition, it provides further insight on the tight regulation of transposable elements.
However, I think the paper has a series of weaknesses.*
*1. MXB is an* *Interferon (IFN) stimulated gene and in most cells, is virtually absent in the absent of IFN stimulation. On the other hand, LINE-1 has been linked to type 1 IFN induction before. However, the authors fail to make this connection. Is LINE-1 inducing an IFN response and then upregulating MXB? Or is this mechanism (LINE-1 inhibition) only valid for cells/tissues with some basal level of MXB? An easy experiment to link MXB to the inhibition of LINE-1 would be adding IFN to a CRISPR control cell line and to the MXB KO lines and quantify the extent of the IFN inhibition, where it should be reduced for the MXB KO lines.*

**Response:** We have now measured the effect of interferon on LINE-1 retrotransposition in the control and MxB knockout cells, and observed partial loss of interferon inhibition of LINE-1 in MxB knockout cells, supporting the role of MxB in interferon-mediated inhibition of LINE-1. The results are presented in Fig S2H, described on page 6 lines 118 to 123, “IFN has been shown to inhibit LINE-1 replication (44). Since MxB is an IFN stimulated gene, we investigated the role of MxB in IFN-mediated LINE-1 inhibition. Activity of CMV-L1-neo^RT^ in the control and MxB knockout cells was measured with and without IFN. The results of colony assay showed that LINE-1 retrotransposition was strongly inhibited by IFN in the control cell line, but this inhibition was significantly lost in MxB knock-out cell line (Fig. S2H), which indicates that IFN-induced MxB contributes to IFN inhibition of LINE-1.”

*2. Authors claim that nuclear envelope localization of MXB is an important determinant for the LINE-1 inhibitory mechanism. In the case of HIV-1, several articles have linked the importance of nuclear pore proteins to the MXB antiviral activity. I am missing some evidence on this regard. Are any nuclear pore proteins involved in LINE-1 inhibition by MXB? Are any nuclear pore proteins located to the MXB-containing stress granules?*

**Response:** We have now examined the nuclear pore complex protein NUP214 and importin TNPO1 which have been reported to interact with MxB and are required for anti-viral function of MxB. The results are described on page 12, lines 242 to 251, “It was reported that MxB is localized at cytoplasmic face of nuclear core complex (NPC) and interact with NPC protein NUP214, as well as transport reporter transportin-1 (TNPO1) (47, 64). We thus investigated whether NUP214 or TNPO1 which are required for LINE-1 inhibition by MxB. We observed that TNPO-1, but not NUP214, was localized to the MxB/ORF1p cytoplasm foci (Fig. S8C and S8D). When we knocked down endogenous NUP214 or TNPO1, we found that NUP214 knockdown led to moderate decrease of LINE-1 retrotransposition, whereas TNPO-1 knockdown moderately increased LINE-1 retrotransposition (Fig. S8E and S8F). Knockdown of neither NUP214 nor TNPO-1 changed MxB inhibition of LINE-1. These data suggest a modest role of NUP214 and TNPO-1 in LINE-1 retrotransposition, but neither of these two proteins affect MxB inhibition of LINE-1”

*3. Authors claim that MXB and ORF1P accumulates to stress granules and that transient depletion of G3BP1 or TIA1 depletes MXB of its inhibitory activity. I would like to see further confirmation of this. Specifically, will depletion of G3BP1 and/or TIA1 affect the accumulation of ORF1P and MXB to cytoplasmic bodies? I don’t think figures 4e and 4f really show the formation of stress granules.*

**Response:** We have now knocked down G3BP1 or TIA1 with siRNA in HeLa cells stably expressing MxB-GFP, then transfected with CMV-L1-Neo^RT^ reporter DNA. Results of immunofluorescence experiments show that depletion of either G3BP1 or TIA1 reduces cytoplasmic ORF1p/MxB granules (Fig. S6C and F). The results are described on page 10, lines 197 to 219 “Knockdown of endogenous G3BP1 or TIA-1 led to the loss of ORF1p/MxB-EGFP granules in the cytoplasm (Fig. S6C and S6F), further supporting the stress-granule nature of the ORF1p/MxB foci.”

*4. I am not convinced by the microscopy throughout the paper, specifically:*

*a. Fig 2: MXB distribution should be the same in the EGFP panel for the MXB L1 NEO condition of fig 2a and the MXB-EGFP + L1 NEO panel from S3a, and in the Flag panel for the MXB-Flag condition in fig 2c. However, there is no MXB located to the nuclear envelope in fig 2c, while this is clearly noticeable in fig 2a and S3a.*

**Response:** We have now repeated the experiment in Fig. 2C using the LINE-1-ms2x6/MS2-GFP two plasmids system to visualize the subcellular localization of LINE-1 RNA which contains six MS2-binding sites. MxB-Flag was detected using anti-Flag antibody and pseudocolored as red, and was co-localized with LINE-1 RNA (in green), in agreement with the co-localization of MxB-GFP with ORF1p shown in Fig. 2A and 2B. Localization of MxB to nuclear envelope is less evident when co-localized with ORF1p or LINE-1 RNA as shown in Fig. 2A (in green), Fig. 2B (in green) and Fig. 2C (in red).

*b. Fig 6C: again, I am not convinced by this figure. None of the nuclear envelope-located proteins seem to be locating there. Staining with MXB and/or nucleoporins creates a tight ring around the nucleus (see for example Kane et al., 2018), while in here there is some space between the DAPI signal and the Flag signal, as if between the nucleus and the nuclear envelope was some “empty” space. In the case of human MXB there is not even such a ring, but just aggregation of protein in the proximity of the nucleus.*

**Response:** We have repeated the experiment more carefully and presented the representative images of human MxB-Flag in Fig. 6C. Human MxB-Flag shows localization to the nuclear envelope.

*c. Lines 196-197: “in agreement with the inability of these MxB mutants to interact with G3BP1 or ORF1p, (Fig. S6A and B)”. This is not what I understand from S6a and S6b. Firstly, if I am not mistaken, in S6a colocalization of MXB, G3BP1 and L1 is indicated by the light-pink colour on the merge image. While this might be absent in the case of del25 and the M574D/Y651D mutant, I can see it in the K131A and R449D/F495D mutants.*

**Response:** We agree that some of the MxB mutants did display co-localization with G3BP1/ORF1p but at a lower degree compared with the wild type MxB. We have revised this sentence to, “in agreement with the decreased association of these MxB mutants with G3BP1 or ORF1p (Fig. S9A and B now)” (please see page 12, lines 256-257.)

*5. Figure legends are too succinct. Some of the figures are virtually impossible to understand looking only at the legend, in others some graphics are not explained (as is the case of the arrows in microscopy images) and in most of them further explanation is needed to understand what they are showing.*

**Response:** We have now provided greater details in the figure legends to allow better understanding of the data shown in the figures.

*Minor comments*

*1. Author summary needs some correction, number of grammatical mistakes and apparent lack of text, such as:
a. Line 30: “Retrotransposons have been existed as ancient components”.
b. Line 32: “and inserts in genome by its endonuclease, which staggers”.
c. Line 35: “Among* *the risk of LINE-1 out of control, host develops”.
d. RNP is not defined*

**Response:** We have now thoroughly revised the author summary and corrected the grammatical mistakes. “RNP” denotes “ribonucleoprotein”, on page 2, line 39.

*2. Line 70: HBV, a reference is missing*

**Response:** The reference (34) about HBV has been added on page 4, line 78.

*3. Fig S1: It is not clear to me how the colony assay is quantified. It is not specified in M&M (where it probably should be), and in the legend of fig S1 it reads “Number of neomycin-resistant colonies with the control vector is arbitrarily set as 1”. I assume authors mean set as 100. Nevertheless, in figures depicting this assay (such as S1b and 1a &1b), the average of the vector control is not 100 (in S1b for example is clearly above it). Taking again S1b as an example, it seems that only one of the dots has a value of exactly 100, while the other 2 are clearly higher. Does this mean that one of the vector control experiments was set as a 100, and then all the other results (for vector and MX proteins) normalized to this value, rather than on each experiment setting the vector control to 100 and then normalizing the MX proteins to it?*

**Response:** We have now provided a detailed description of the LINE-1 colony assay. The actual colony numbers have now been shown in the relevant figures including Fig. 1A, 1B, 4D, 4E, 5C, 5D, 6A, 6B and 6F. We have also revised the figure legends in Fig. S1.

*4. Fig S1d doesn’t have a legend. In addition, -globin is misspelled as, -glubin.*

**Response:** We have now added the legend to the Fig. S1E (original Fig. S1D). The globin correction has been made.

*5. Fig S2: The order of the different panels should be modified: S2e is presented in the text before any other panel from S2.*

**Response:** We have now revised Fig. S2E as Fig. S2A.

*6. S2b: the level of MXB in both lines seems the same to me. One of the ref given on the text for previously showing presence of MXB in HeLa cells in the absence of IFN doesn’t show that, on the contrary, there is a total absence of MXB in the no IFN lane (see fig 1g from ref 36).*

**Response:** HeLa cells have modest basal expression of MxB in the absence of IFN treatment, which becomes visible in Western blots with longer exposure, as described in ref 42, “HeLa cells appear to have modest MxB levels that become more apparent upon longer exposures (Supplementary Fig. 4, PMID: 32102993)”. We have repeated the Western blots and now presented clearer endogenous MxB signals in Fig, S2C and E.

*7. S2d: immunblot lacks enough quality to draw any conclusions from it.*

**Response:** We have now repeated the Western blots and present the data as Fig. S2E.

*8. Line 123: a “was” is missing.*

**Response:** We have corrected this mistake.

*9. Line 129: experiments instead of experiment.*

**Response:** We have corrected this mistake.

*10. Fig 2H: wouldn’t be expected an increased signal for L1ORF1 in the cytoplasm as the nuclear accumulation is reduced under MXB expression?*

**Response:** We didn’t observe the increase of LINE-1 ORF1p in the cytoplasm under MxB expression. One explanation is that ORF1p is mainly located in cytoplasm, and the reduction of its amount in the nucleus does not cause significant change to its cytoplasmic level.

*11. Fig 2I: I fail to see any signal for L1ORF1 in the nucleus.*

**Response:** To clearly observe the endogenous L1ORF1p signals, we have repeated the Western blots with greater amounts of samples, and presented the results in Fig. 2I.

*12. Fig 2j: why is there less ORF1 p positive cells when expressing EGFP than when expressing MXB-EGFP?*

**Response:** Thank you for pointing out these images that do not represent the high-content screen results shown in the bar graph. As YFP (26.2 kDa) tag is bulky for ORF1p, we have now repeated these experiments with an ORF1p bearing the Flag tag and presented the representative images in Fig. 2J.

*13. Fig 3a: shouldn’t the level of L1ORF1 been reduced under MXB expression? This was shown in fig 1c.*

**Response:** We have repeated the experiment to confirm that endogenous ORF1p was reduced under MxB expression. We presented more representative images in Fig 3A.

*14. Fig 3b: the intensity of the* *H2AX staining needs to be increased. It is quite difficult to see it as it is now.*

**Response:** We have now changed the pseudocolor of H2AX to allow clear visualization of H2AX signals in Fig. 3B.

*15. Fig 3e: needs more intensity.*

**Response:** We have now changed the pseudocolor of H2AX to make the fluorescence signals more visible.

*16. Fig 3g: I am not convinced by these results. First, what is mock here? No explanation in the figure legend. Second, why is there a marked increase of H2AX from mock to EN- if the level of ORF1P is the same? Third, I do see a small reduction of the H2AX signal in the EN- under MXB expression, is this statistically different from the reduction seen in the EN+ condition?*

**Response:** As this reviewer noticed, the EN-/EN+ system has very low level of ORF1p expression and retrotransposition activity. We have therefore introduced the 261/262RR to AA mutation into ORF1 which was shown to inactivate CMV-L1-neo^RT^ retrotransposition (*Moran et al. Cell 1996),* and repeated the experiment of H2AX activation with the mutated and wild type CMV-L1-neo^RT^. The results are described on page 9, lines 171-172 “Ectopic MxB decreased the γH2AX level induced by CMV-L1-neo^RT^ (Fig. 3G)”.

*17. Fig 4a and 4b: the arrows shown are not mentioned in the figure legend, what are they signalling?*

**Response:** The arrows indicate the localization of ORF1p in nucleus, this is now described in the figure legend (Fig. 4A and 4B).

*18. Fig 4b: why is the distribution of ORF1P more diffuse in the absence of MXB (also seem in S4a)? This is not the case in Fig 4a.*

**Response:** We suspect that this is because MxB interaction with ORF1p causes the formation of MxB/ORF1p-containing SGs, thus creating the more punctate feature of ORF1p distribution, compared with the relatively more diffused ORF1p in the absence of MxB.

*19. Fig 4c: Why is MXB not localize to the nuclear envelope here?*

**Response:** It is most likely that MxB is more co-localized with LINE-1 RNA protein complex, thus has less at the nuclear envelope.

*20. Lines 152-153: it reads “Indeed, the MxB/ORF1p foci had G3BP1 and TIA1 in HeLa cells that stably expressed MxB and were transfected with CMV-L1-neoRT reporter (Fig. 4A)”. However, TIA1 staining is not shown.*

**Response:** We have now presented co-localization of TIA1 with MxB/ORF1p in HeLa cells that stably expressed MxB and were transfected with CMV-L1-neo^RT^ reporter or ORF1p, and described the results on page 9, lines 183 to 184, “Indeed, the MxB/ORF1p foci contained G3BP1 and TIA1 in HeLa cells that stably expressed MxB and were transfected with CMV-L1-neo^RT^ reporter or ORF1p plasmid (Fig. 4A, 4B, S5A and S5B)”. We also present the co-localization of TIA1 with transiently expressed MxB and ORF1p in Fig. S6D and E, and described the data on page 9, lines 185 to 187, “The co-localization was also observed in HeLa cells that were transiently transfected with MxB plasmid DNA (Fig. S6A, S6B, S6D and S6E).”

*21. S4a: figure labelling contains a MXBE instead of MXB.*

**Response:** We have corrected the “MxBE” label as “MxB-EGFP” in Fig. S6A (original Fig S4A).

*22. Fig 4d: it would enrich the paper seeing also the interaction with TIA1.*

**Response:** We have performed the co-IP experiments and observed interaction of TIA1 with MxB/ORF1p (shown in Fig. 4D).

*23. Line 185: MXB-B25 should be MXA-B25 or even MXA+B25, to keep consistency with line 181.*

**Response:** We have made the correction to MxA+B25.

*24. Fig 5a: there is no explanation for the MXB domains labelling.*

**Response:** We have described MxB domains in the legend of Figure 5.

*25. Fig 5b: why is MXA aggregating in the cytoplasm? Previously it has been reported as a diffuse cytoplasmic protein (see for example Goujon et al., 2014 or Betancor et al., 2019) and it behaves this way in fig 6c. Also, none of the proteins with a nuclear envelope localization signal (MXB and MXA+B25) or an NLS (MXBdel25 + NLS) seems to accumulate at the nuclear envelope or at the nucleus.*

**Response:** We have now tested different amounts of plasmid DNA used in transfection, and when less plasmid DNA was used, did observe subcellular distribution of Mx proteins as this reviewer indicated. We have therefore now presented the new confocal microscopy data in Fig. 5B and 5E.

*26. In previous works, experiments using MXA chimaeras bearing the N-terminal domain of MXB and showing transfer of the antiviral activity have used MXA proteins where the whole N-terminal domain of MXA has been substituted with the whole N-terminal domain of MXB (see for example Goujon et al., 2014). I would recommend doing this experiment rather than attaching the first 25 amino acids of MXB to MXA, since this could potentially interfere with the function of the N-terminal domain.*

**Response:** We have generated the new MxA+B25 protein by replacing the first 42 amino acids of MxA with the first 25 amino acids of MxB, and measured MxA+B25 expression with Western blot and IF. The results are described on page 12, lines 237 to 239, “Interestingly, the MxA+B25 protein, which has its first 42 amino acids changed to the first 25 amino acids of MxB, was localized to the peri-nuclear region, and did not inhibit LINE-1 (Fig. 5B and C)”.

*27. Line 194: should say mutant M574D/Y651D instead of M574D, Y651D*

**Response:** We have corrected this mistake.

*28. Lines 194-195: since the authors are describing mutants, they should state they are using the* *R449D/F495D mutant, instead of saying they also mutate those 2 positions, since in the current format the text is not clear on whether they created 2 independent mutants or a double mutant (as they did).*

**Response:** We now use “R449D/F495D” mutant on page 12, line 254.

*29. Fig S6: I found confusing the arrangement of the figure. It seems like both bar charts at the bottom belong to S6d. The lack of any mention to the quantification of the immunoblots from S6c makes it more confusing. A further explanation on the figure legend is required.*

**Response:** We have now added in figure legend, “The bar chart at the right of S9C (Original S6C) represents the quantification data of the immunoblot.”

*30. The IP showed in S6b doesn’t show the lack of interaction of MXB mutants with L1ORF1: there is a clear signal for the latter in all the lanes and in the case of the vector control. Better experiments are needed to be able to make this claim.*

**Response:** We have repeated more this IP experiment to reduce the background MxB signals in the vector control, and did observe interaction of MxB with LINE-1 ORF1p, whereas MxB mutants virtually lost this interaction (shown in Fig S9B now).

*31. Line 225: what are IAP and MusD?*

**Response:** We have now provided their full names, and described on page 14, lines 287 to 288, “IAP (Intracisternal A-type Particle elements) and MusD are active mouse ERVs (endogenous retroviruses) (also named LTR (long terminal repeat) retrotransposons) (64).”

*32. Fig 6e: colour code description of the bars is missing in the figure.*

**Response:** We have removed bar colors to avoid confusion.

*33. Lines 257-258: “MxB inhibition of LINE-1 is CypA-independent (data not shown)”. While ideally such a claim should be accompanied by the data showing this, authors could further explained how they found this (CRISPR KO?, siRNA? Use of cyclosporine?, etc)*

**Response:** We have now presented the effect of CypA knockdown on LINE-1 retrotransposition in Fig S8 A and B. and described the data on pages 10 to 11, lines 214 to 219, “To answer whether CypA affects the MxB inhibition of LINE-1, we used shRNA of CypA to knock down endogenous CypA in HeLa cells and performed colony assay of cells transfected with MxB and CMV-L1 neo^RT^ reporter DNA. The results showed that LINE-1 retrotransposition was inhibited by MxB as much in CypA knockdown cells as in the control cells (Fig. S8A and S8B), which indicates that MxB does not require CypA to restrict LINE-1 retrotransposition.”

*34. Line 288: remove “in” at the end of the line.*

**Response:** We have corrected this mistake.

*35. Line 289: a space is missing before “(65)”. Also see point 45.*

**Response:** We have corrected this mistake and proofread the whole text.

*36. Line 303: “Equus caballus Mx2, but not Ovis aries Mx2, inhibits LINE-1”. This claim is not supported by data in Fig 6B, since there are less colonies in cells expressing Ovis MX2 than in cells expressing MXA (with statistical significance).*

**Response:** As this reviewer pointed out, the Ovis aries Mx2 did suppress LINE-1 activity, although the effect is weaker compared to that by human and Equus caballus Mx2. We have now revised the description on page 18, line 377-378, “Equus caballus Mx2 strongly inhibited LINE-1, whereas the Ovis aries Mx2 exhibited modest inhibitory effect.”

*37. Line 320: a “the” is missing in “D205A point mutation in endonuclease”.*

**Response:** We have added “the”.

*38. Lines 321-322: the sentence “IAP-neoTNF, MusD-neoTNF reporter plasmids which contain mouse LTR retrotransposon and reverse direction neomycin resistance cassette (19)” is missing something*

**Response:** We have revised this sentence as, “IAP-neo^TNF^ and MusD-neo^TNF^ reporter plasmids contain mouse LTR retrotransposon and a neomycin resistance cassette in reverse orientation.” (Please see page 19, lines 404 to 405)

*39. In general, the “plasmids and antibodies” part of M&M needs rewriting since there are several grammatical mistakes.*

**Response:** We have revised the “plasmids and antibodies” section.

*40. Line 325: the mutation is M574 not N574*

**Response:** We have corrected this mistake.

*41. Line 326: how is the MXB-EGFP generated? Is EGFP at the N or C-terminal end of MXB?*

**Response:** EGFP was fused at C-terminus of MxB. This is now described on page 19 to 20, lines 410 to 412, “The MxB sequence was inserted to the N-terminus of EGFP in pEGFP-N1 vector between the cleavage sites of restriction enzymes BamH1 and Age1, to generate MxB-EGFP fusion protein.”

*42. Line 333: is missing “antibody” at the end.*

**Response:** We have corrected this mistake.

*43. Line 335: is also missing antibody after “(05-1938)”*

**Response:** This mistake has now been corrected.

*44. Line 336: is Millipore*

**Response:** We have corrected this mistake.

*45. Throughout the text there are many spaces missing between the text and the reference call.*

**Response:** We have now added this missing space throughout the text.

*46. In “cell colony assay” an explanation on how the results are normalized is missing.*

**Response:** we have now presented the G418-resistant cell colony numbers in figures, to avoid the confusion caused by arbitrary normalization.

*47. The way the number of hours is depicted is not consistent throughout the text. For example, in line 358 it says “18 hours”, while* *in line 367 it says “seventy-two hours”*

**Response:** We have now used Arabic numerals.

*48. Line 401: the “1,000” is the only time authors used a “,” all the other 1000 are without it. Also, some other numbers throughout the text are depicted with or without “,”. Consistency is needed.*

**Response:** We have revised all the number depiction to ensure consistency.

*49. Line 412: “were” should be “was”*

**Response:** This has been corrected.

*50. The M&M are in general too summarized. In most cases the amount of DNA transfected or the time after transfection is not indicated.*

**Response:** We have now provided more details in the “M&M” section including the amount of plasmid DNA used in transfections.

*51. Line 432: “Na3VO4” should be written as Na3VO4.*

**Response:** This has been corrected.

*52. Line 439: Should say “by centrifugation” instead of “with centrifugation”*

**Response:** This has been corrected.

*53. Line 443: What viruses?*

**Response:** We have now removed “viruses”.

***Reviewer #2:***

*In their manuscript “MxB inhibits Long Interspersed Element 1 retrotransposition”, Huang et al. demonstrate that the cellular antiviral protein MxB blocks L1 retrotransposition in cultured cells, and then investigate the mechanism by which this inhibition occurs using cultured cell retrotransposition assays, immunofluorescence imagining, and biochemistry approaches. Over all, the manuscript presents results that will be interesting to the L1 and virology fields. However, some claims are over-stated particularly with regards to the mechanism of inhibition, and some of the experiments could be presented more clearly particularly in the figures and legends.*

*Major points:*

*1. MxB was demonstrated to restrict L1 retrotransposition by Goodier and colleagues in 2015 (PMID 26001115). This publication is mentioned in the discussion of the present manuscript, but should be mentioned in the abstract or introduction. Otherwise the manuscript gives the initial impression that L1 inhibition by MxB is a novel finding. In addition,* *Doucet et al. (PMID:* *20949108) used epitope-tagging strategies to investigate the subcellular localization of L1 proteins and RNA. This work should be cited.*

**Response:** We have now cited the work by *Goodier and colleagues in 2015 (PMID 26001115)* on page 4, lines 67 to 70, “In 2015, Goodier and colleagues screened a panel of ISGs and restriction factors with known antiviral activities for effects of their expression on L1 retrotransposition. They demonstrated that BST2, ISG20, MAVS, MX2, and ZAP showed strong L1 inhibition (28). However, the mechanism of MxB inhibiting LINE-1 has not been completely elucidated.”

We have also cited *Doucet et al. (PMID: 20949108)* when we described the LINE-1-ms2x6/MS2-GFP system on page 7, line 146, cited ref (46).

2 *While the results as a whole demonstrate that MxB mediates the localization of L1 ORF1p to stress granules, the conclusion that MxB inhibits L1 specifically by preventing L1 RNPs from entering the nucleus is not sufficiently supported. Figure 2 is titled with this claim, but the immunofluorescence images in A-C do not convincingly or quantitatively show ORF1p localizing to the nucleus in the absence of MxB, and previous studies have shown that wild-type ORF1p does not normally localize to the nucleus (**PMID* *29309036,* *PMID* *20949108, PMID* *20147320). Figure J claims to show this quantitatively using IF, but YFP (26.2 kDa) is a very bulky tag and is likely to affect cellular localization of ORF1p (40 kDa). This experiment would be much stronger if it utilized a smaller epitope tag already demonstrated to minimally impact L1 retrotransposition (eg, T7--PMID 20949108, PMID 16183655)*

**Response:** We agree that LINE-1 ORF1p is mainly detected within the cytoplasm, yet we did observe ORF1 foci in the nucleus, which is in agreement with the report by Nita et al (PMID:29309036). This latter study also showed that leptomycin B treatment, which blocks Crm1-medited nuclear export, increased levels of nuclear ORF1. The nuclear presence of ORF1 assists reverse transcription of LINE-1 RNA by ORF2p. We also examined ORF1 in the cytoplasm and nucleus fractionation experiment, and observed the decrease of nuclear ORF1 with MxB expression.

To avoid the effect of the bulky YFP tag on the subcellular distribution of ORF1p, we repeated the high-content image experiments using the ORF1-Flag protein. The results showed significant reduction of nuclear ORF1p in MxB-expressing cells (Fig. 2J).

*3 The fractionation experiment in panels H and I is a bit confusing: in H, the amount of ORF1p found in the nucleus with vector-only is dramatically more than in the cytoplasm, but this pattern is not supported by the immunofluorescence experiments in panel A. The quantitation shown in panels H and I should be better explained—the actual ratio of ORF1p in nucleus/cytoplasm for vector control, for example, is clearly not 1. How was this normalized? In addition, in panel I the detection of endogenous ORF1p in HeLa cells is not supported by previous studies, which describe HeLa cells to express very little or* *no ORF1p (PMID* *20686575). Over all, the claim that MxB prevents nuclear import of L1 RNPs should be softened.*

**Response:** The strong signal of MxB in the nuclear fractions is because we loaded more nuclear fraction samples in order to detect the low-level nuclear MxB, this is now added in the M&M section, on page 25, lines 531 to 532, “To visualize nuclear ORF1p, we examined in the Western blots nuclear fraction samples from cells 5 times more than the cytoplasmic fraction samples.”

We also described in the M&M section on page 25, lines 533-537, “To calculate the ratio of nuclear/cytoplasmic MxB, we first normalized the amount of nuclear ORF1p with the nuclear LAMN protein level, the amount of cytoplasmic ORF1p with tubulin, then nucleus/cytoplasm ORF1p ratio was calculated by dividing nuclear ORF1p value by the cytoplasm ORF1p value. At the end, the nucleus/cytoplasm ORF1p ratio of the vector control is arbitrarily set at “1”.”

Endogenous ORF1p is indeed expressed at low levels, although higher in certain cell types such as embryonic stem cells than others such as HeLa (PMID: 20686575). Endogenous LINE-1 retrotransposition in HeLa cells was previously reported (PMID: 16107723). We were able to detect endogenous ORF1p in HeLa cells thanks to the antibody- 4H1 mouse monoclonal antibody from Millipore (catalog number MABC1152). Garcia-Perez and colleagues used a rabbit polyclonal anti-ORF1 antibody (a generous gift from Dr Thomas Fanning) in their study to detect endogenous ORF1p (PMID: 20686575).

We have now revised the conclusion on MxB preventing LINE-1 RNP nuclear import, please see page 2, lines 25 to 27, page 15, lines 313 to 315, “MxB sequesters LINE-1 RNP within the cytoplasm, thus prevents LINE-1 RNP from accessing the nucleus to complete reverse transcription.”

*4 In the paragraph beginning with line 100, the authors say that the reduced detection of L1 RNA and ORF1p is consistent with MxB inhibiting retrotransposition. The rationale for this statement should be clarified—if the authors are proposing a sequestration model (rather than an inhibition of L1 expression), one would not necessarily expect the L1 RNA and protein levels to be affected.*

**Response:** When ORF1p and L1 RNA were detected in MxB stably expressed cell lines or MxB knockout cell lines, after passages, the ORF1p and RNA were declined. However, there was no decline of LINE-1 ORF1p in transient transfected MxB situation (Fig S1B). After many generations of MxB stably expressed or knockout, MxB sequestrated LINE-1 RNP in SGs may also lead to decrease of ORF1p and LINE-1 RNA levels.

*5 Throughout the manuscript, the figure legends and labels, particularly on panels showing western blots, could be clearer and more informative. For example, many IF images have white arrows but it is never stated what these indicate. In figure 3A, the gel segment labeled “EGFP” shows detection in the MxB-EGFP transfection but not in the EGFP alone transfection. Presumably this is because the molecular weights of MxB-EGFP and EGFP alone are different, but this should be clarified. The figure legend for supplemental figure 1D is missing entirely.*

**Response:** We proofread the whole manuscript, figure legends and labels, and provided more information to make them more comprehensible. Particularly, we have now provided more details in figure legends including the legend for Fig 1D (Fig. 1E now) and description of the arrows in the relevant IF images. We have also repeated the Western blots in Fig 3A and included the EGFP bands.

*6 Throughout the paper, sequestration of ORF1p by MxB is only shown by IF for exogenously expressed ORF1p. Given that the authors appear to have an antibody that apparently can detect endogenous ORF1p by western blot, it would greatly strengthen the paper to demonstrate that MxB sequesters endogenous ORF1p via immunofluorescence.*

**Response:** We did use the ORF1p antibody to detect endogenous ORF1p in MxB-EGFP stably expressing cells and MxB knockout cells by IF. However, endogenous ORF1p is difficult to detect with IF, even though it is detectable by western blot.

*Minor comments
1. Line 54: cite a reference for the number of human genetic disease cases attributed to L1-mediated mutagenesis.*

**Response:** The following two references are cited on page 3, line 56, “17. Suarez NA, Macia A, Muotri AR. LINE-1 retrotransposons in healthy and diseased human brain. Dev Neurobiol. 2018;78(5):434-55.

18. Payer LM, Burns KH. Transposable elements in human genetic disease. Nat Rev Genet. 2019;20(12):760-72.”

*2. Line 69: Would be informative to discuss in more detail the mechanism(s) by which MxB and MxA inhibit viruses.*

**Response:** We have now described more how MxA and MxB inhibit viruses on page 4, lines 73 to 80, “MxA is cytoplasmic protein, inhibits a plethora of viruses from diverse families, including influenza A virus, vesicular stomatitis virus, LaCrosse virus, hepatitis B virus and others (30). The antiviral activity of MxA relies on the GTP binding and hydrolysis domain, oligomerization of the stalk domain and the intact BSE (bundle signaling element). In contrast, much fewer viruses have been reported to be restricted by MxB. These viruses include HIV-1 (31-33), HBV (34), HCV (35) and herpesviruses (36, 37). MxB is mainly observed at the nuclear envelope, inhibits HIV-1 and herpesviruses through targeting viral capsid and blocking nuclear import of viral DNA (38).”

*3. Line 86: state quantitatively what the “lowest amount” of plasmid was.*

**Response:** It is 200 ng of plasmid DNA, please see page 5, line 98.

*4. Figure 6D: which mouse L1 retrotransposition constructs were used? No papers cited and not shown in methods.*

**Response:** We did not use mouse L1 retrotransposition in Figure 6D, we measured human CMV-L1-neo^RT^ reporter activity in the BALB/3T3 clone A31 cell line which stably expressed human MxB-flag. In the experiments shown in Fig. 6E and 6F, two mouse LTR retroelements IAP and MusD were tested. We have cited the reference (65) on page 14, lines 283 to 290.

***Reviewer #3:***

*In this manuscript, Huang et al. invested the mechanism of MxB-mediated LINE-1 suppression. They started by confirming this reported phenomenon in a neomycin-resistance-based LINE-1 retrotransposition assay. Then IF and IP experiments were conducted, results of which suggested that MxB and LINE-1 ORF1p co-localizes in some dot-like organelles, which were later determined as stress granules. The authors further demonstrated the MxB suppresses LINE-1 replication through stress granule pathway, because shutting down expression of SG-associated proteins such as G3BP1 and TIA1 significantly compromises MxB’s efficiency in LINE-1 inhibition. Tests with mutated MxB indicated that nuclear membrane localization, oligomerization, and GTPase activity are all essential for MxB to suppress LINE-1. At last, the authors presented evidence suggesting that only some members of the Mx protein family are effective in LINE-1 regulation, and MxB can suppress other retrotransposons such as murine LINE-1, IAP, and MusD.*

*Taken together, this study suggested that changing the subcellular localization of ORF1p in host cells is the key mechanism for MxB-mediated LINE-1 suppression. And combining with previous reports, restricting ORF1p onto SG and/or p-bodies might be a common mechanism shared by various LINE-1 suppressors. Such information is both interesting and important for the LINE-1 community, and might shed light on future research in the Genetics and Genomics field. However, I am not fully convinced because some conclusions in this manuscript were not fully supported, while some phenomena were not fully investigated.

Major issues:

1. I first found it impressive that the authors tested MxB through three different systems: knockout, stable expression, and transient expression. And then I found myself confused because at most times it was difficult to find out which system the authors used for a statement in the text or a panel in a figure. For instance, the authors performed IP experiments in Fig. 2D, E, and F, but there was no description whether the experiments where performed in MxB stably expressed or transiently expressed HeLa cells. It would be nice if the authors clearly state which system was used when describing data in the text.*

**Response:** We have now clearly indicated in the main text and the figure legends which MxB expression method was used for the specific experiment, including the co-IP experiments that were all performed with transiently expressed MxB-Flag. We described “(D, E, F) Co-immunoprecipitation of transiently expressed MxB-Flag and ORF1p which was expressed either from the ORF1p-Myc DNA (D), the CMV-L1-neo^RT^ DNA (E), or the endogenous LINE-1 in HEK293T cells (F).” (Fig. 2 legend)

*2. Some of the statements were not logical. In Line 121, the authors stated, “The association of MxB with LINE-1 RNA…”, while no LINE-1 RNA detection was performed with MxB interactants. The interaction between ORF1p and MxB cannot guarantee the binding between MxB and LINE-1 RNA. Because it is possible that, by binding ORF1p, MxB can sabotage the interaction between ORF1p and LINE-1 RNA. Besides, Fig. 2G needs improvement, as the ORF1p bands in the IP samples are hard to see, esp. when comparing to previous results in Fig. 2D, E, and F.*

**Response:** The sentence “The association of MxB with LINE-1 RNA…” has now been revised to, “The association of MxB with LINE-1 RNP was also supported by the data of co-immunoprecipiation experiments performed with ectopic ORF1p (Fig. 2D and 2E) or endogenous ORF1p (Fig. 2F).” (Please see page 8, lines 147 to 149. In addition, we have now presented the co-IP data with clearer ORF1p bands in Fig 2G.

*3. The use of Fig. S7 is also puzzling. Though it separates different Mx proteins from different species, the result had no impact on the selection of Mx proteins for subsequent LINE-1 retrotransposition assay. My best guess is that the authors chose some Mx proteins from each branch for subsequent tests. If true, the authors should elaborate the reason(s) in more details. Also, the procedures for the reconstruction of the phylogenetic tree was not mentioned. In addition, the tree should be* *bootstrap tested, with bootstrap values (>70) shown on the nodes of the tree to indicate the credibility. There is only one panel in Fig. S7, so the panel letter “A” is not necessary.*

**Response:** We have now described the method constructing the phylogenetic tree and provided accession number for each Mx gene. Bootstrap values (>70) were also calculated and presented in Fig. S10 (the original Fig S7). Results of poylogenetic analysis guided us to select MxA-like and MxB-like proteins for the IF imaging and LINE-1 inhibiting experiments. We have now removed the “A” label.

*4. Some of the experiments were poorly designed and/or presented. For example, the authors should at least show one negative result for neomycin selection in Fig. S1B where the Neo-based LINE-1 retrotransposition assay is first presented. Such a negative result can be generated by transfecting HeLa cells with any retrotransposition-incompetent vectors (such as pJM111) reported by Moran et al. (Cell 1996), and can help to confirm LINE-1 retrotransposition events shown in Fig. S1B.*

**Response:** We have now mutated 261/262RR to AA in ORF1 to generate a retrotransposition-deficient L1, and described L1-ORF1(RR261/262AA) in Materials and Methods on page 19, lines 402 to 403, “The L1-ORF1 (RR261/262AA) plasmid was generated by inserting ORF1 261/262RR to AA mutations into the CMV-L1-neo^RT^ plasmid, as reported in (40).” The results of colony assay are presented in Fig. S1B.

*5. Why no elevation of ORF1p expression could be detected in cells transfected with LINE-1-EN- or LINE-1-EN+ in Fig. 3G?*

**Response:** As this reviewer indicated, we also noticed that the EN-/EN+ system expressed very low level of ORF1p. We have therefore switched to the CMV-L1-neo^RT^ system and generated a mutated L1 which has the RR261/262AA mutation in ORF1p as reported in the pJM111 plasmid used by *Moran et al. Cell 1996,* which is not capable of retrotransposition. We repeated the experiment in Fig 3G and described at page 9 line 171-172 as “Ectopic MxB decreased the γH2AX level induced by CMV-L1-neo^RT^ (Fig. 3G).”

*6. Lines 149-154: The authors mentioned TIA1 here but no TIA1 images were shown in Fig. 4A and B.*

**Response:** We have now added TIA1/MxB/ORF1p subcellular localization results in Fig. S5A and 5B either with transfected CMV-L1-neo^RT^ reporter or ORF1p. We also knocked down endogenous G3BP1 or TIA1, and observed decrease in the MxB/ORF1p co-localization (Fig S6C and S6F).

*7. Which experiment was conducted to generate Fig. 6E? And why some of the “relative mRNA levels” were not set as 1 for vector (control) groups?*

**Response:** We now describe on page 14, lines 283 to 285 as “Levels of endogenous mouse LINE-1 subfamilies (L1A, L1Gf and L1Tf) and SINE RNA were measured by RT-qPCR with promoter specific primers in BALB/3T3 clone A31 cells that stably express human MxB.” We have set the value for each LINE-1 or SINE in the vector control as 1.

*8. Fig. S6B is not acceptable. The ORF1p band detected in the IP eluant of the negative control (i.e., the vector group) significantly compromises the interpretation of the whole IP results. The authors should repeat this experiment to achieve a better image as they did in Fig. 2D-F.*

**Response:** We have now repeated the IP experiment and presented the new data in Fig S9B (original Fig S6B).

*9. And the investigation for observed phenomena was not thorough in some cases. In Lines 100-108, the authors suggested that by suppressing LINE-1 retrotransposition, MxB has an impact on the expression of ORF1p. However, the authors completely neglected the possibility that MxB might induce the depletion of ORF1p, even when they later found that MxB can interact with ORF1p. Such an idea can be verified simply through Western blotting tests on cells transfected with ORF1p and MxB expressing vectors.*

**Response:** When ORF1p and L1 RNA were detected in MxB stably expressed cell lines or MxB knockout cell lines, the ORF1p and RNA were declined (Fig 1 C and G). However, there was no decline of LINE-1 ORF1p in transient transfected MxB situation (Fig S1B). After many generations of MxB stably expressed or knockout, MxB sequestrated LINE-1 RNP in SGs may also lead to decrease of ORF1p and LINE-1 RNA levels. It’s an accumulation effect.

*10. In Lines 165-171, the authors concluded that “MxB is located to G3BP1- and TIA1-bearing foci through interaction with ORF1p.” This also means the presence of ORF1p also altered the subcellular distribution of MxB which normally associates with nuclear membrane. The authors should test it to confirm, as such a phenomenon might interrupt other functions of MxB.*

**Response:** We agree that ORF1p may change the subcellular localization and hence the other cellular functions of MxB. This possibility is now discussed on page 17, lines 347 to 349, “In retrospective, sequestration of MxB to stress granules as a result of interaction with ORF1p alters subcellular localization of MxB, and may potentially influence its other cellular functions.”

*11. MxB suppressing murine SINE, IAP, and MusD is another example. Although it is impressive to known that MxB has a wider spectrum in regulating retroelements, these data introduce confusion to the study. To suppress LINE-1, MxB alters the subcellular localization of ORF1p. If ORF1p is an important component for the replication of murine SINE, IAP, and MusD, the authors should state it clearly, with proper references. If not, then the authors should investigate or at least speculate (with reasonable facts) whether MxB suppresses these retroelements through similar mechanisms.*

**Response:** We have now discussed how MxB may suppress murine SINE, IAP, and MusD retroelements on page 18, lines 386 to 390, “SINE does not encode proteins, is unable to retrotransposition autonomously, its activity depends on LINE-1 ORF1p and ORF2p (14, 15). MxB is thus expected to inhibit SINE through sequestering LINE-1 ORF1p as we observed in this study. IAP and MusD are endogenous retroviruses, they encode Gag protein as HIV-1 does (71). It is likely MxB targets Gag and inhibits IAP and MusD, similar to MxB inhibiting HIV-1 by targeting viral capsid.”

*12. In Discussion, the authors should provide possible reason(s) why the GTPase activity is essential for MxB-mediated LINE-1 suppression, or ORF1p re-localization.*

**Response:** We have now discussed this on pages 17 to 18, lines 367 to 371 “Our data showed that the GTPase mutation K131A prevents MxB localization to the nuclear envelope and impairs its interaction with ORF1p. Furthermore, Betancor and colleagues reported that the GTPase domain cooperates with the N-terminal domain in Mx2 binding to the HIV-1 capsid (61). We speculate that the GTPase activity may influence MxB conformation which is involved in interaction with LINE-1 ORF1p (Fig. S9C) and inhibiting LINE-1 retrotransposition (Fig. 5D).”

*Minor issues:

Text:

1. Full name for LINE-1 should be corrected to "long interspersed element type 1". No "nuclear" in the name. This convention dates back to early eighties (see Singer MF, Cell 1982). “L1” is also ok, which was used in the manuscript (e.g., L1-neo) but the authors failed to explain what “L1” stand for.*

**Response:** We now change to “long interspersed element 1 (LINE-1, L1)” in the manuscript.

*2. The authors used both “ORF1” and “ORF1p” to refer the open reading frame encoding the 40kDa protein. Be consistent. And please notice that “ORF1p” with the small p in most LINE-1-related literatures indicates the protein but not the ORF.*

**Response:** We have now used “ORF1p” to refer ORF1 protein and used “ORF1” to refer the open reading frame in the correct context.

*3. In Line 46, the authors stated, “LINE-1 is still active”, which can be misleading. Among ~500,000 LINE-1 copies in one single cell, only a very small fraction of them are competent in retrotransposition (this has been mentioned in many LINE-1 research papers and reviews). In addition, the word “active” is confusing, as many LINE-1 copies, though incompetent in replication, are active in transcription.*

**Response:** We have revised this sentence to, “Among ~500,000 LINE-1 copies in human genome, about 80 to 100 LINE-1 elements are still capable of retrotransposition.” (Please see page 3, lines 47 to 48)  ***4****. In Line 63, the authors stated, “digesting LINE-1 DNA by TREX-1”, while Li et al. reported that the exonuclease activity is not involved in TREX1-mediated LINE-1 suppression; instead, TREX1 inhibits LINE-1 by inducing the proteasome-mediated proteolysis of ORF1p (NAR 2017).*

**Response:** We have now revised this statement on page 3, lines 64 to 67, “These host factors operate by targeting LINE-1 RNP, and either directly degrading LINE-1 RNA by RNase L, mutating newly synthesized LINE-1 DNA by APOBEC3 proteins, degrading ORF1p by TREX-1, or sequestering LINE-1 RNP in the cytoplasmic granules by MOV10, SAMHD1 or ZAP.”

*5. What did the authors mean by “LINE-1 DNA production” in Line 88?*

**Response:** We have now changed “LINE-1 DNA production” to “LINE-1 DNA level” on page 5, line 101.

*6. Line 149: I believe the word “APOBC” here should be “APOBEC”.*

**Response:** This mistake has been corrected.

*7. The following statements need to be referenced: Line 53: Not surprisingly,…and Alu insertion.*

**Response:** The references (14) and (15) have now been added on page 3, line 54.

*8. Line 70: HBV(ref) (I believe the authors meant to insert references here but forgot).*

**Reference:** This missing reference has now been added, on page 4, line 78.

*Figure:

1. Some of the figure legends are sloppy and confusing. For example, the description for Fig. 3G focused on tested HeLa cells, while the panel actually shows Western blotting results. The authors should correct such errors.*

**Response:** We have proofread the figure legends throughout the manuscript and added more details. Particularly, we have revised the figure legend of Fig. 3G.

*2. Fig. 2A, B, and C: Transfected vectors should be shown clearly by each set of images. Also, “LINE-1-MS2” in Fig. 2C is not a proper description, as green fluorescence can only indicate the localization of MS2-GFP, but not its status for LINE-1 RNA binding.*

**Response:** We have re-labeled Fig. 2A, 2B and 2C to clearly indicate the expression vectors used. For Fig. 2C, we have changed label of L1-MS2 to L1-ms2x6, and described in Figure legend, “(C) Immunofluorescence microscopy of MxB and L1-ms2x6/MS2-GFP. The transcribed LINE-1 RNA bears six copies of the MS2-binding sites which can be bound with MS2-GFP to indicate the localization of LINE-1 RNA.”

*3. Fig. 2H and I: explain what the letters “C” and “N” stand for. And I suggest that the space between panel H and I should be increased. In current form, it is hard to determine whether the label “L1-Neo” belongs to H or I.*

**Response:** We have increased the space between Fig 2H and 2I. We have also described C and N in the legend.

*4. Bar charts in Fig. 2H, I, and J, and Fig. S6: the titles of y-axes should be changed, as it is not difficult to determine which protein has been targeted for nucleocytoplasm distribution.*

**Response:** We have revised the Y-axis title as “Relative ratio of nuclear/cytoplasmic ORF1p”.

*5. Fig. 4A and B: was it MxB or MxB-EGFP used in these tests?*

**Response:** MxB-EGFP was used in the experiment, as now described in the Fig. 4 legend, “Co-localization of stably expressed MxB-EGFP with ORF1p and G3BP1 in HeLa cells. ORF1p was either expressed from the transfected CMV-L1-neo^RT^ DNA (A) or ORF1p vector DNA (B).” And we also changed label in Fig. 4A and B with “MxB-EGFP”.

*6. Fig. 6D: what is “huMaB” that was shown in the bar chart?*

**Response:** “huMaB” is corrected to “huMxB”.

*7. Explain those arrows used in Fig. 2A, 2B, 4A, 4B, S3, and S4.*

**Response:** These arrows were used to indicate nuclear ORF1p. This is now described in the figure legends.

*8. In CMV-LINE-1-neoRT (the authors needs to confirm whether it is the pJM101 plasmid used in* *Moran et al. Cell 1996), there is a CMV promoter next to the 5’ side of LINE-1 5’-UTR. The authors missed it in Fig. S1A.*

**Response:** We have now added the missing CMV promoter to 5’ side of LINE-1 5’-UTR in Fig. S1A, and also described this in the legend.

*9. In the figure legend for S1B, it was stated that, “Number of neomycin-resistant colonies with the control vector is arbitrarily set as 1”. However, the y axes of bar charts in S1B (and other LINE-1 assay results) are showing numbers of colonies. And it should not be labeled as “colony assay” in the bar chart, which is a test but not a result. A proper title for the y-axes in bar charts showing neomycin-resistance-based L1 assay results can be found in Herrmann et al. Mol DNA 2018.*

**Response:** We have now revised the figure legend and changed the title of Y-axis to “G418-resistant foci”.

*10. There is no description in the figure legend for Fig. S1D.*

**Response:** Apology for this oversight. We have now added the legend for Fig. S1E (original Fig. S1D), “HEK293T cells were co-transfected with the CMV-L1-neo^RT^ DNA and MxB DNA. Levels of the newly synthesized LINE-1 DNA were determined by semi-quantitative PCR. Levels of β-globin DNA were measured as internal controls. Intensities of DNA bands were quantified, the results are summarized in the bar graph.”

*11. Fig. S4A: What is “MxBE”?*

**Response:** We have corrected “MxBE” to “MxB-EGFP”.

*12. Explain the difference between Fig. S6D and the bar chart next to it (on the right)*

**Response:** We have now re-organized this figure (now Fig. S9) to clearly indicate that the bar chart (originally next to Fig. S6D) is the quantification result of Fig. 9C, the relative ratio of nuclear/cytoplasmic ORF1p. Fig. S9D presents the result of high-content image data, this is described in the legend.

***Reviewer #4:***

*The present study from Haung et al. investigates the anti-viral restriction factor MxB as also an inhibitor of LINE-1 retrotransposons. Some data reported here duplicates that of a previous report by Goodier et al. (2015, PLoS Genetics) including that MxB (but not MxA) decreases L1 retrotransposition, that MxB associates with L1 ORF1p in an RNA-dependent manner, and that the K131 MxB mutation which affects GTP binding restores retrotransposition. (These confirmatory data should be noted as such).

New data include demonstrating increase in L1 retrotransposition and endogenous L1 RNA and protein in an MxB KO cell line, decrease in L1 RNA and protein in a MxB knock-in cell line, and that MxB associates with the L1 RNP in cytoplasmic stress granules. Furthermore, the authors report that MxB reduces the amount of nuclear vs cytoplasmic ORF1p with a consequent reduction in H2AX-detected DNA damage, and that inhibition of stress granule components limits MxB inhibition of the L1. Thus, this study expands considerably on the previous limited work and proposes a mechanism for the effect of MxB on LINE-1s. As such it is worthy of consideration for publication in PLoS Genetics. However, while detailed supporting data has already been presented, I believe some additional controls and experiments are required to more strongly support the interesting claims made in this paper.

MAJOR:
1. There are no obvious specifically stated toxicity and* *cell growth rate assays for potential effects of MxB in transfected and knock-out cell lines. These should be shown. The N1-EGFP transfection control (p. 4, line 83) might be considered as an toxicity assay.*

**Response:** We have now performed the cell proliferation assay and cell viability assay, presented the data in Fig. S2F and S2G, described on page 6, lines 111 to 113 as “In addition, we measured the viability of MxB knockout cells and found no significant difference between MxB knockout and control cell lines (Fig. S2F). The same observations were made with cells stably expressing MxB (Fig. S2G).”

*2. p. 4, line 111 (also abstract, p. 11, line 240, p. 12, line 248). The section title and conclusion, "MxB sequesters LINE-1 ORF1p in cytoplasmic bodies" is not necessarily correct. If this is claimed, the numbers of cells with ORF1p in SGs in the presence or absence of overexpressed MxB should be counted. It is stated on p. 13, line 272 that "we observed much greater LINE-1 ORF1p localization to G3BP1/MxB-positive cytoplasmic granules compared to the relatively more dispersed cytoplasmic distribution of ORF1p in the absence of MxB expression ((Fig. 4A, B and S4A, B)." If so this needs to be backed up by cell count quantification. In our experience overexpressed EGFP-ORF1p forms many stress granules in numbers of unstressed cell lines, including HeLa cells****.***

**Response:** We agree with this reviewer that expression of LINE-1 ORF1p itself can form stress granules, which has also been observed in our study. We have scored the number of ORF1p-containing stress granules with and without MxB expression, observed no significant difference between the stable MxB-expressing cell line and control cell line. However, we observed larger ORF1p-positive cytoplasmic foci which contain G3BP1/MxB compared with control cells, and ORF1p displayed more diffuse distribution without MxB over-expression. We therefore propose that MxB promotes the sequestration of ORF1p to G3BP1/TIA1-positive cytoplasmic foci, thus enhancing the stress granule-based mechanism of restricting LINE-1. (Please see our discussion on page 16, lines 343 to 350)

***3****. Furthermore, on p. 8, line 165 (also p. 14, line 291) it is noted that ectopic or endogenous MxB does not form arsenite-induced SGs in the absence of transfected ORF1p. That would suggest that overexpressed ORF1p sequesters MxB in SGs rather than the other way around. However, Fig. S3A, second row shows MxB-EGFP alone forming many SGs; furthermore, in the fourth row there are a significant number of ORF1p SGs that do not contain MxB protein -- so this is confusing regarding the authors' conclusion.*

**Response:** This reviewer raised an important point, we have discussed this on page 17 line 359-365, “MxB is not an integral component of stress granules, since we did not observe MxB localization with stress granules that were induced with arsenite treatment (Fig. S7A and B). Expression of MxB itself did not cause stress granule formation either. We suspect that MxB association with ORF1p stimulates G3BP1/TIA1-dependent aggregation of ORF1p, either through the GTPase activity of MxB and/or the ability of MxB to oligomerize. This mechanism allows MxB to inhibit LINE-1, also sends MxB to the ORF1p/G3BP1/TIA1 positive cytoplasmic granules.” To reflect this discussion, we have revised the subtitle “MxB sequesters LINE-1 ORF1p in cytoplasmic bodies” to “MxB promotes sequestration of LINE-1 ORF1p to cytoplasmic bodies” on page 7, lines 135 to 136.

*4. The definitive experiment is to see in the MxB KO cell line vs its wild-type control if overexpressed and endogenous ORF1p forms fewer stress granules/in fewer cells (both unstressed and arsenite-stressed). If the above claim is to be made, these experiments should be done. The monoclonal 4H1 anti-ORF1p antibody from* *Millipore is a good one; I believe it is available in China.*

**Response:** We now describe on page 10, lines 207 to 212, “We treated both the control and MxB knockout cell lines with AS. The number of SGs increased significantly with arsenite treatment, yet there was no difference between MxB knockout and control cell lines (Fig. S7C). When the CMV-L1-neo^RT^ plasmid was transfected, the number of ORF1p-containing SGs did not differ between MxB knockout and control cell lines with or without arsenite induction (Fig. S7D). These results suggest that arsenite -induced ORF1p-containing SGs is not affected by endogenous MxB expression.”

*5. Fig. 3, F. This is unclear to me: are the controls and KO cell lines transfected with the L1? If not, is there any data for transfected cells?*

**Response:** We have described in the legends of Fig. 3F (also 3D and 3E) that no LINE-1 was transfected. We also performed H2AX foci experiments in CMV-L1-neo^RT^ transfected MxB knockout cell line, the results are presented in Fig. S4, described on pages 8 to 9, lines 170 to 171, “When LINE-1 DNA was transfected into MxB knockout cell lines, γH2AX level and foci number increased compared with control cells.”

*6. Also, Fig. 3G, and p. 7, line 142. It is stated in the text that, "As a control, the endonuclease inactivated LINE-1 (EN-) did not increase γH2AX foci, and MxB overexpression had no effect (Fig. 3G)." No cell counts are presented in this figure to show the effect of the EN- control on γH2AX foci production: these should be shown. Also it is stated, "As a control, the endonuclease inactivated LINE-1 (EN-) did not increase γH2AX foci". However, there is a significant increase in γH2AX protein in both EN- and EN-transfected cells, which seems contradictory. Quantification of foci numbers could help in clarification.*

**Response:** The LINE-1 EN-/EN+ system expresses very low level of ORF1p and is inefficient in retrotransposition. We thus introduced the RR261/262AA mutation into ORF1 as described in (40) to generate an inactive CMV-L1-neo^RT^, and used this L1 mutant as a control in the experiments examining H2AX activation. The results are now presented in Fig. 3G, and the inactive L1 did not increase H2AX expression.

***7. p. 8, line 169. Also, it is concluded "Together, these data demonstrate that MxB associates with LINE-1 RNP in the stress granules, thus prevents nuclear import of LINE-1 proteins and diminishes LINE-1 retrotransposition." The authors are implying that MxB sequesters ORF1p in SGs to prevent further function. However, as noted above, to me the converse appears true, that ORF1p sequesters MxB.***

**Response:** We have now revised this statement, on page 10, lines 205 to 207, “Together, these data demonstrate that MxB associates with LINE-1 RNP in the stress granules, and enhances cytoplasmic sequestration of LNE-1 RNP.”

*8. Fig. 4D. The IP of G3BP1 by MxB is not entirely convincing due to background presence of MxB in the other two lanes. Also, there seems to be somewhat more G3BP1 in the MxB lane compared with vector. Can a better blot be shown? Quantification of band intensities might also help.*

**Response:** We have repeated this IP experiment and presented the new data in Fig. 4D. We have also blotted the presence of ORF1 and TIA1. The data showed a very low level of G3BP1, TIA1 in the vector control.

*9. p. 9, line 186. There is a discrepancy with previous findings. In the present study, MxBdel25 lost the ability to inhibit retrotransposition while Goodier et al. reported that deleting the first 25 aa of MxB had no effect on retrotransposition (although their K131A mutation did, in agreement with the present study, a fact that should be also mentioned). This discrepancy should be noted and discussed. Perhaps epitope tags are having some effect.*

**Response:** We have discussed this discrepancy on page 16, lines 326 to 329, “We also noted that the N-terminal sequence was also reported to be dispensable for MxB to inhibit LINE-1 (28). This discrepancy with our observation may be a result of the different experimental systems used in different studies including cell lines and protein tags. More studies are warranted to resolve this discrepancy.*”*

*MINOR.
1. p. 4, line 87. I point out that possible artifacts of quantitative PCR to measure levels of LINE-1 integrated cDNA in cells have been proposed in the literature, including generation of non-integrated ectopic cDNA by L1 RT which would then be amplified by PCR and misinterpreted as increased genomic insertions. I would suggest removing the word from '...to measure levels of LINE-1 integrated cDNA in cells".*

**Response:** As suggested, we have removed the word “integrated” to reflect the fact that this PCR protocol measures the total level of LINE-1 DNA.

*2. Please note what arrows in all figures indicate. Some appear to show foci overlapping nuclei which is confusing.*

**Response:** We have described in figure legends what these arrows indicated (please see Fig. 2A, 4A, S3A, S3B, S5A, S6A, S6B, S6D and S6E). ***3****. Fig. S2D. The quality of the Western showing loss of MxB protein in KO cells is poor. Can a better one be presented?*

**Response:** We have now presented a clearer blot for MxB in Fig. S2E (original Fig S2D).

*4. Fig. 4E,F. Is the apparent increase in retrotransposition seen by knock-down of TIA-1 or G3BP1 significant? If so, this would appear worthy of comment in the text.*

**Response:** We have now described on page 10, lines 193 to 197 “Results of LINE-1 reporter assays showed that MxB lost inhibition of LINE-1 when either G3BP1or TIA1 was knocked down (Fig. 4E and F).” We also observed 50% increase in LINE-1 retrotransposition with knockdown of either G3BP1 or TIA1 in the absence of MxB expression (Fig. 4E and F) (26).”

*5. p. 7, line 144. It is noted, "These data further suggest that MxB inhibits the nuclear import of LINE-1 RNP composed of LINE-1 RNA, ORF1p and ORF2p." While it is often assumed, I believe it has never been proven that ORF1p enters the nucleus bound to the L1 RNA. This caveat could perhaps be noted.*

**Response:** We have now revised this sentence to, “These data further suggest that MxB reduces the formation of double-strand DNA breaks associated with LINE-1 retrotransposition.” (Please see page 9, lines 173 to 174)

*6. Fig. 6E, p. 11, line 223. It needs to be made clearer in the text that these mouse elements were detected by RT-PCR. Where were the primers located in the L1s?*

**Response:** We have described the PCR method to detect mouse LINE-1 on page 14, lines 283 to 285, “Levels of endogenous mouse LINE-1 subfamilies (L1A, L1Gf and L1Tf) and SINE RNA were measured by RT-qPCR with promoter specific primers in BALB/3T3 clone A31 cells that stably express human MxB.”gen

*7. p. 12, line 257. It is noted, "MxB inhibition of LINE-1 is CypA-independent (data not shown)". Show the data please if you wish to state this.*

**Response:** We have now presented the CypA knockdown data on LINE-1 inhibition by MxB in Fig. S8A and B.

*8. There are a number of quite minor English grammar errors scattered through the manuscript, a majority involving missing articles.*

**Response:** We have proofread the manuscript and corrected the grammar errors.
