# Supplementary material for: MxB inhibits long interspersed element type 1 retrotransposition
Source: PLoS Genet. 2022 Feb 16;18(2):e1010034. doi: 10.1371/journal.pgen.1010034 (PMC8849481; doi:10.1371/journal.pgen.1010034)
Supplement: Attachment — Submitted filename: respones to reveiwer-PGENETICS-D-21-01057 R2.docx [file pgen.1010034.s013.docx]

**Reviewer #1:**

**I find this version of the manuscript greatly improved. Authors have addressed most of my comment and results obtained are consistent. However, I still have some concerns about some microscopy images and with the explanation of some results:**

**1. Lines 40-41: the sentence “This is the first report attributing the restriction function of MxB to sequestering LINE-1 RNP, likely viral RNPs, to stress granules” is incorrect. Do authors mean like viral RNPs? If so, I think is still not correct, to the best knowledge of this reviewer, there is not evidence that cytoplasmic MxB is the one driving viral inhibition (certainly not in the case of HIV-1)**

Response: We have now revise this statement to, “Hence our data attribute the restriction function of MxB to sequestering LINE-1 RNP to stress granules.” at page 2, lines 27 to 28.

**2. It would be advisable to include a blot showing MX2 in Ctr and KO lines in Fig 1D rather than going to sup Fig 2E. This would ease interpretation of the Figure**

Response: We have now presented this MxB immunoblot in Fig 1D.

**3. Sup Fig 3A. Have the authors quantified the colocalization of ORF1p with GFP (in both, EGFP and MxB-EGFP)? Just by looking at the merge pictures is difficult to tell if colocalization is higher with MxB-EGFP than with EGFP (Look at EGFP granules specially)**

Response: Co-localization of EGFP or MxB-EGFP with ORF1p was analyzed with fluorescence intensity analysis software from LAS AF (Leica) and shown in Fig. S3A.

**4. Line 151: “These results suggest that MxB associates with LINE-1 RNA and ORF1p” I am not convinced by this statement. Since there is no direct evidence of MxB interacting with LINE1 RNA, I think authors can’t rule out the diminish interaction in the presence of RNase due to a conformational change of ORF1p, different oligomerization status, etc, rather than because MxB directly binds the RNA**

Response: We agree with the alternative scenarios to interpret the data, and have revised this statement as, “These results suggest that MxB associates with ORF1p in an RNA-dependent manner.” at page 8, lines 151-152.

**5. Line 163: should say ORF2p**

Response: This has been corrected.

**6. Line 172: defective, not “detective”**

Response: This has been corrected.

**7. Lines 163-174 and Fig 3: I am confused by this experiment. Cells depleted of MxB show an increase in γH2AX protein level and associated foci. Does this mean that MxB is a down modulator of γH2AX expression and its absence results in γH2AX uncontrolled expression? Furthermore, the number of γH2AX foci in EGFP expressing cells transfected with L1-neo is comparable to the one seeing in MxB KO cells (specially clone 2). I think these results need further explanation**

Response: MxB expression indeed suppresses the level of γH2AX which assembles at the sites of damaged DNA (which can be caused by LINE-1 retrotransposition) and initiates DNA repair process. We have revised the description of these data on page 9, lines 174 to 176, “We also observed that MxB overexpression alone diminished the number γH2AX foci, and MxB knockout itself led to an increase in γH2AX foci, which is likely a result of the suppression of endogenous LINE-1 by MxB.”

**8. Line 197: an “in” is missing before agreement**

Response: This has been corrected.

**9. Line 203: a “cell” is missing after HeLa**

Response: This has been corrected.

**10. Line 206: It says “LNE” instead of LINE**

Response: This has been corrected.

**11. Sup Fig 8B: why is the number of G418 resistant colonies reduced in the shCypA + vector condition compared to NC?**

Response: We have now stated on page 9, lines 226 to 228, “Fewer G418-resistant cell colonies were scored with CypA-knockdown, which indicates a positive role CypA in LINE-1 retrotransposition.”

**12. Line 223 onwards: I think it would help to notice somewhere in this paragraph that MxBdel25** **is the same as the natural** **short isoform of MxB**

Response: We have now stated on page12, lines 242 to 243 “the distribution was the same as the natural short isoform of MxB (64).”

**13. Line 243: TNPO1 is transport receptor transportin 1, not transport “reporter”**

Response: This has been corrected.

**14. Line 244: “which” should be removed**

Response: This has been corrected.

**15. Fig 5: Shouldn’t MxBdel25+NLS be located inside the nucleus? In fig 5b it seems to localize just as MxA.**

Response: This is what has been expected, yet MxBdel25+NLS is not found in the nucleus as also reported by other groups (PMID 25348155, Engelman et al).

**16. Sup Fig 9: localization of MxBdel25 in panel A doesn’t recapitulate image shown in 5E. It seems as there was no signal for GFP and what is shown is background (including signal in the nucleus).**

Response: We agree that the MxBdel25 signal in Sup Fig 9 is quite weak, we have now used images with stronger MxBdel25 signal.

**17. Lines 326-329: Discussion would be enriched if authors also mention the importance of the N-terminal domain of MxB for HIV-1 inhibition, involving capsid binding (****Fricke et al., 2014), or regulation by phosphorylation (****Betancor et al., 2021)**

Response: We have now added on page 16 lines 335 to 338, “It has been reported that MxB N-terminal sequence participates in the recognition of HIV-1 capsid structure and this activity is subject to regulation by phosphorylation (71, 72). It is possible that MxB interaction with LINE-1 ORF1p also requires its N-terminal sequence.”

**18. Line 371: remove ” at the end of line.**

Response: This has been corrected.

**19. Lines 384-390: I think the authors should discuss the fact that MxB is able to inhibit retrotransposons in mice cells, which points to either MxB acting alone, or being** **assisted by human and murine proteins indistinctively.**

Response: We have now discussed this on page 19 lines 403 to 405, “Since MxB also inhibits retrotransposons in murine cells, it can be envisioned that MxB either exerts its inhibition by itself or is assisted by factors shared by human and murine cells.”

**20. Line 780: “show”, not showed**

Response: This has been corrected.

**21. It’s my opinion figure legends should include what the error bars represent (SEM, SD…) or the statistical test used, to ease their interpretation.**

Response: We have been added that the error bars represent SEM and what statistical test method has been used, in figure legends.

**22. Line 837: “non-human” instead of “no-human”**

Response: This has been corrected.

**23. Line 843: “endogenous” instead of “endogenouse”**

Response: This has been corrected.

**24. Line 844: “grey”, not “gray”.**

Response: This has been corrected.

**Reviewer #2:**

**Responses to major points:
1. Goodier et al and Doucet et al have now been appropriately cited.**

**2. The authors cited relevant literature to counter that ORF1p does not normally localize to the nucleus. They have repeated the immunofluorescence assay in Figure 2J using a smaller tag to detect ORF1p, and achieved a similar result to ORF1p-YFP.**

**3. The authors explain that the strong nuclear signal for MxB in the fractionation experiment in panels 2H and I is due to loading 5x more nuclear fraction than cytoplasmic fraction in their Western blot. They have put this information in the Materials and Methods, but I think it needs to be either in the main text or in the figure legend. I also think the explanation for how the normalization was done in panel 2I belongs in the figure legend, not the materials and methods. These experimental details are critical to interpreting the work.**

Response: We have been added these fractionation experiment details in the legends of Fig. 2H and I.

**4. I am still unclear on why sequestration of ORF1p and LINE-1 RNA in stress granules by MxB1 would lead to an over-all decrease in ORF1p and L1 RNA levels.**

Response: One explanation is that, after prolonged storage in stress granules, LINE-1 RNA is degraded.

**5. The figure legends are much more clearly over all, and I appreciate that the Western blot in 3A has been repeated to show the relevant control bands.**

**6. I appreciate that it is difficult to detect endogenous ORF1p by immunofluorescence. I don't think it is critical to do so for the paper.**

**All minor points have been adequately addressed. I appreciate in particular the background on how MxA and MxB inhibit viruses**

**Reviewer #3:**

**By adding more data and explanations, the manuscript by Huang, et al. has been greatly improved for its logic, clarity, and significance. All my previous concerns have been addressed. However, with the ms revised, the authors also introduced some typos and gramma errors.

Here are some typo examples:

Line 94: RR261/261AA should be RR261/262AA**

Response: This has been corrected.

**Line 206: LNE-1 should be LINE-1**

Response: This has been corrected.

**Lines 242-251: Both TNPO-1 and TNPO1 were used in this paragraph. Be consistent.**

Response: We have now used TNPO-1 throughout the manuscript.

**Line 371: The quotation mark should be removed.**

Response: This has been corrected.

**Line 416: The authors should check these cat.#: 66009-I-Ig and 67447-l-lg, as they were purchased from the same company and share similar numbering pattern but use different letters (the authors may set the font as “Times New Roman” for better display).**

Response: “66009-I-Ig and 67447-l-lg” have been corrected to “66009-1-Ig and 67447-l-Ig”.

**Line 470: I believe the “****RNA free water” here should be “RNase free water”.**

Response: This has been corrected.

**Lines 549-562: The authors should notice that some accession numbers listed in this paragraph were underlined.**

Response: We have removed the underline.

**Reviewer #4**

**Huang and group have made considerable efforts to improve the manuscript. While I am generally satisfied with the revised manuscript as it relates to my concerns, there are a couple of continuing points.

1. The authors now report to the reviewers, "We have scored the number of ORF1p-containing stress granules with and without MxB expression, observed no significant difference between the stable MxB-expressing cell line and control cell line" -- this new data should be noted in the Results section. They go on to note that they instead observed larger ORF1p-positive cytoplasmic foci. How was this enlargement of foci determined? I assume by visible inspection (rather than software analyses) -- this should be stated in the text and the number of cells examined to give this impression noted.**

Response: We have now described these data on page 19, lines 189 to 191, “We have scored the number of ORF1p-containing stress granules with and without MxB expression, observed no significant difference between the stable MxB-expressing cell line and control cell line (Fig. 4A, 4B, S5A and S5B).”

We have also stated on page 9, lines 191 to 192, “However, we observed the enlarged ORF1p-containing foci under MxB-GFP overexpression by visually inspecting more than 100 cells”.

**2. Furthermore, on page 10 line 197 it is stated, " [In] agreement with these functional data, knockdown of endogenous G3BP1 or TIA-1 led to the loss of ORF1p/MxB-EGFP granules in the cytoplasm (Fig. S6C and S6F), further supporting the stress-granule nature of the ORF1p/MxB foci." If this statement is made, it needs to be quantitated with counts in the figure.**

Response: We have scored the number of ORF1p/MxB-EGFP granules in more than 50 cells and presented that results in in Fig. S6C and S6F. The details have been included in the figure legend.

**3. The authors comment, "This reviewer raised an important point, we have discussed this on page 17 line 359-365, “MxB is not an integral component of stress granules, since we did not observe MxB localization with stress granules that were induced with arsenite treatment (Fig. S7A and B). Expression of MxB itself did not cause stress granule formation either." However, this statement remains inconsistent with Fig. S3A,** **second row, which still shows MxB-EGFP alone forming many SGs. Does this mean endogenous MxB does not enter SGs, but that ectopic overexpression of MxB can induce SGs. I am willing to believe that, but these matters need to be clarified in the text.**

Response: Overexpressed MxB is often seen to form aggregates in the cytoplasm, but these aggregates are not marked by stress granule markers.

**4. Fig. 2A and B. White arrows are claimed to show ORF1p foci in the nuclei that disappear with MXB expression. While it has previously been reported that ORF1p enter nucleoli and can form foci in nuclei of some cells (****Pereira et al. 2018 Mob. DNA), are the authors certain these foci are truly in the nucleus and not cytoplasmic foci imaged on top of nuclei?** **Z-stack analysis would confirm this and the point should be clarified in the Figure text.**

Response: We did not record Z-stack images of MxB, thus cannot perform Z-stack analysis. In our confocal imaging settings, the Z-axis of the image is 600 nm thick, which is far less than the 7 μm thickness of HeLa cells. More importantly, we often chose the largest diameter of the nucleus for recording, a position which is far away from the top of the nucleus. And the nuclear ORF1p foci were observed in multiple cells.

**5. Fig. 3G. The figure panel still shows the same Western as before for EN- and EN+. Please change to now show the WT and ORF1 mutant modified data.**

Response: We have presented the new data of WT and ORF1 RR261/262AA in Fig. 3G, please see below the new data and the original data.

**6. In the Materials and Methods section the title "Quantification of integrated LINE-1 DNA by PCR" also needs to be changed to "Quantification of LINE-1 cDNA by PCR" as was done in the results section.**

Response: This has been corrected.
